# Targeting integrin αvβ3 with indomethacin inhibits patient‐derived xenograft tumour growth and recurrence in oesophageal squamous cell carcinoma

**DOI:** 10.1002/ctm2.548

**Published:** 2021-10-28

**Authors:** Fangfang Liu, Qiong Wu, Wei Han, Kyle Laster, Yamei Hu, Fayang Ma, Hanyong Chen, Xueli Tian, Yan Qiao, Hui Liu, Dong Joon Kim, Zigang Dong, Kangdong Liu

**Affiliations:** ^1^ Department of Pathophysiology School of Basic Medical Sciences China‐US (Henan) Hormel Cancer Institute AMS, College of Medicine Zhengzhou University Zhengzhou China; ^2^ China‐US (Henan) Hormel Cancer Institute Zhengzhou China; ^3^ Hormel Institute University of Minnesota Austin Minnesota USA; ^4^ State Key Laboratory of Esophageal Cancer Prevention and Treatment Zhengzhou China; ^5^ Provincial Cooperative Innovation Center for Cancer Chemoprevention Zhengzhou University Zhengzhou China; ^6^ Cancer Chemoprevention International Collaboration Laboratory Zhengzhou China

**Keywords:** ESCC, indomethacin, integrin αvβ3, recurrence

## Abstract

**Rationale:**

A high risk of post‐operative recurrence contributes to the poor prognosis and low survival rate of oesophageal squamous cell carcinoma (ESCC) patients. Increasing experimental evidence suggests that integrin adhesion receptors, in particular integrin αv (ITGAV), are important for cancer cell survival, proliferation and migration. Therefore, targeting ITGAV may be a rational approach for preventing ESCC recurrence.

**Materials and methods:**

Protein levels of ITGAV were determined in human ESCC tumour tissues using immunohistochemistry. MTT, propidium iodide staining, and annexin V staining were utilized to investigate cell viability, cell cycle progression, and induction of apoptosis, respectively. Computational docking was performed with the Schrödinger Suite software to visualize the interaction between indomethacin and ITGAV. Cell‐derived xenograft mouse models, patient‐derived xenograft (PDX) mouse models, and a humanized mouse model were employed for in vivo studies.

**Results:**

ITGAV was upregulated in human ESCC tumour tissues and increased ITGAV protein levels were associated with poor prognosis. ITGAV silencing or knockout suppressed ESCC cell growth and metastatic potential. Interestingly, we identified that indomethacin can bind to ITGAV and enhance synovial apoptosis inhibitor 1 (SYVN1)‐mediated degradation of ITGAV. Integrin β3, one of the β subunits of ITGAV, was also decreased at the protein level in the indomethacin treatment group. Importantly, indomethacin treatment suppressed ESCC tumour growth and prevented recurrence in a PDX mouse model. Moreover, indomethacin inhibited the activation of cytokine TGFβ, reduced SMAD2/3 phosphorylation, and increased anti‐tumour immune responses in a humanized mouse model.

**Conclusion:**

ITGAV is a promising therapeutic target for ESCC. Indomethacin can attenuate ESCC growth through binding to ITGAV, promoting SYVN1‐mediated ubiquitination of ITGAV, and potentiating cytotoxic CD8^+^ T cell responses.

## Graphical abstract

Integrin αvβ3 was upregulated in ESCC tumour tissue compared to adjacent tissue. Indomethacin binds to ITGAV and promotes SYVN1‐mediated ubiquitination of ITGAV, thereby attenuating the αvβ3/PI3K/AKT/GS3Kβ signalling pathway. Inhibition of αvβ3 by indomethacin treatment blocks TGFβ/SMAD2/SMAD3 signalling and increases anti‐tumour immune responses in a humanized mouse model. Indomethacin suppresses ESCC tumour growth and recurrence in a PDX mouse model.

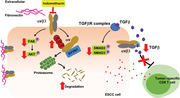

## 1 INTRODUCTION

Oesophageal squamous cell carcinoma (ESCC) is a highly heterogeneous disease that accounts for 80% of oesophageal cancers (ECs)^1^ and produces variable clinical outcomes.^2^ Surgical resection, chemotherapy, or chemoradiotherapy are often used to treat ESCC.^3^, ^4^ However, curative second‐ or later‐line chemotherapy drugs specific for ESCC have not been developed.^5^ Moreover, many chemotherapeutic agents exhibit deleterious effects in ESCC patients, producing adverse outcomes, such as secondary leukaemia, bone marrow suppression, and cardiac toxicity.^6^, ^7^ The 5‐year survival rate for ESCC ranges between 15% and 25% due to inadequate therapeutic strategies and post‐operative recurrence.^8^, ^9^, ^10^, ^11^ Therefore, there is an urgent need to investigate novel strategies for preventing post‐operative recurrence in ESCC patients.

Integrins are a family of cell‐surface transmembrane proteins comprised of 18 α‐subunits and 8 β‐subunits.^12^ Integrin αv (ITGAV), the receptor for fibronectin, forms heterodimers with integrin β1, 3, 5, 6 and 8.^13^ Ligand‐integrin binding promotes the activation of many protein kinases (including FAK and SRC) and their respective downstream effectors.^14^, ^15^, ^16^ Several signalling cascades, including the ERK/JNK and PI3K/AKT pathways, are important for cell proliferation, migration, and survival.^17^ Integrins are also known as activators of latent TGFβ.^18^, ^19^ TGFβ has been shown to promote cancer cell growth through regulating SMAD2/3 phosphorylation upon binding with its receptor.^20^, ^21^ Additionally, TGFβ, as an immunosuppressive cytokine, prevents the penetration of tumours by cytotoxic CD8^+^ T cells in the tumour microenvironment.^19^ Integrin heterodimer formation is mainly regulated by α‐subunit synthesis; therefore, regulation of α‐subunits is particularly important.^22^ Several studies have shown that ITGAV is frequently upregulated in various types of cancers, including EC, melanoma, lung cancer, and prostate cancer.^5^, ^17^, ^23^, ^24^, ^25^ To date, clinical trials designed to assess the efficacy of ITGAV inhibitors have produced unsatisfactory results,^25^, ^26^ suggesting a need to identify novel integrin inhibitors.

Indomethacin, a synthetic non‐steroidal indole derivative, is widely used for its anti‐inflammatory and analgesic properties.^27^ In addition, indomethacin has been shown to inhibit colon cancer cell growth^28^ and decrease EC cell viability.^29^, ^30^ Mechanistically, indomethacin is generally thought to reduce cancer risk by decreasing prostaglandin synthesis through COX inhibition.^27^, ^31^ However, indomethacin was also found to increase p53 expression in xenograft‐derived acute lymphoblastic leukaemia cells,^32^ induce apoptosis by upregulating BAX in oesophageal adenocarcinoma cells^33^, and blocking the translation of eIF2α kinase PKR in colon cancer cells.^34^

In the present study, we found that ITGAV was highly expressed in ESCC. Indomethacin binds to ITGAV, promotes SYVN1‐mediated ITGAV ubiquitination, and decreases integrin αvβ3 protein levels in vivo and in vitro. Moreover, indomethacin decreased TGFβ activity and increased cytotoxic CD8^+^ T cell responses. Our study provides insights into the role of ITGAV in ESCC progression and suggests that indomethacin can inhibit ESCC growth and prevent recurrence through targeting integrin αvβ3.

## 2 MATERIALS AND METHODS

### 2.1 Cell lines

The immortalized Shantou human embryonic oesophageal cell line (SHEE) was provided by Dr. Enmin Li (Shantou, Guangdong, China). ESCC cell lines (KYSE30, KYSE70, KYSE140, KYSE150, KYSE410, KYSE450 and KYSE510) were obtained from Chinese Academy of Science Cell Bank (Shanghai, China). ESCC cell lines were expanded in RPMI‐1640 medium supplemented with 10% heat‐inactivated FBS (Gibco, Carlsbad, CA, USA) and 1% penicillin/streptomycin (Sigma‐Aldrich, St Louis, MO, USA) in a 37°C humidified incubator under 5% CO_2_. HEK‐293T cells (ATCC, Manassas, VA, USA) were cultured in DMEM medium. All cell lines used in the present study were cytogenetically identified and authenticated every 6 months. Hcuman peripheral blood mononuclear cells (PBMCs) were obtained from Milestone (Shanghai, China).

### 2.2 Reagents and antibodies

Indomethacin, iRGD, and cilengitide were purchased from MCE (Junction, NJ, USA). MG132 and cyclohexamide (CHX) were obtained from Sigma‐Aldrich (St. Louis, MO, USA). The ITGAV, fibronectin, ubiquitin, integrin β3 (ITGB3), p‐SMAD2 (S225), SMAD2, p‐SMAD3 (S423 and S425), and SMAD3 antibodies were obtained from Abcam (Cambridge, MA, USA). The p‐FAK (Tyr 925), FAK, PI3K p85α, p‐AKT (Ser 473), AKT, p‐GSK3β (S9), GSK3β, cyclin D1, CDK4, CDK6, SYVN1, NEDD4, ITCH, MYC, CBL, CBLB, anti‐Flag, and anti‐HA antibodies were obtained from CST (Beverly, MA, USA). The β‐Actin antibody was obtained from ZSGB (Beijing, China). The p‐PI3K p85α (Tyr 467), ITGA2, ITGA3, ITGA4, ITGA6, ITGA11, ITGB1, ITGB5, ITGB6, and ITGB8 antibodies were obtained from Santa Cruz Biotechnology (Santa Cruz, CA, USA). The Ki67 antibody was obtained from Thermo Scientific (Waltham, MA, USA). Detailed information regarding the antibodies used within this study can be found in the Supplementary data. Plasmids encoding flag‐ITGAV and HA‐ubiquitin were obtained from Addgene (Watertown, MA, USA). Plasmids encoding HA‐SYVN1, MYC‐UB and MYC‐ITGB3 were from YouBio (Hunan, China). Lipofectamine 2000 was obtained from Invitrogen (Shanghai, China).

### 2.3 Immunohistochemistry and haematoxylin–eosin staining

An ESCC tissue array and PDX tumour tissues were used for immunohistochemistry (IHC) staining. The tissue array was obtained from Outdo Biotech (Shanghai, China). Tumour tissues from the in vivo study were fixed with 4% formaldehyde for at least 48 h and then embedded in paraffin blocks before being processed for immunohistochemical analysis. Serial 5‐µm paraffin tissue sections were deparaffinized at 60°C for 2 h and then rehydration using alcohol and PBS. Next, the slides were boiled in sodium citrate buffer solution for 15 min. Afterwards, the tissue sections were treated with 3% H_2_O_2_ for 8 min and then blocked in 10% goat serum for 40 min at 24°C. The slides were subsequently incubated with the following antibodies (Ki67, 1:200; ITGAV, 1:200; ITGB3, 1:150; p‐FAK, 1:150; p‐PI3K, 1:150; p‐AKT, 1:150; p‐GSK3β, 1:200) at 4°C for 12 h. The slides were then washed with PBS and stained with the appropriate secondary antibody. Finally, the slides were stained with diaminobenzidine for 2 min and then counterstained with haematoxylin. Representative fields of each slide were photographed using an inverted microscope. The results were quantified using the Image‐Pro Plus software. For haematoxylin–eosin (HE) staining, PDX tumours were harvested and subjected to 4% paraformaldehyde fixation for at least 48 h, followed by dehydration through an alcohol gradient (100%, 95% and 75%). Tissues were embedded in paraffin blocks and sectioned into 4 µm slices with a microtome. These sections were subjected to incubation with HE reagents.

### 2.4 ITGAV silencing and knockout

An shRNA‐mediated approach was used to generate ITGAV‐knockdown cells. The following lentiviral expression vectors were transfected into HEK‐293T cells using Lipofectamine 2000 Reagent: pLKO.1, shITGAV plus pMD2.G and psPAX2. After incubation for 48 h, the lentivirus‐enriched culture medium was harvested, filtered, and stored at −80°C until required for use. The KYSE30 and KYSE510 ESCC cells were seeded and infected with lentivirus medium supplemented with polybrene (8 µg/ml; Sigma‐Aldrich). After 24 h, the infected cells were selected in complete growth medium supplemented with 4 µg/ml of puromycin (Sigma‐Aldrich) for 24–48 h. Cells were scraped from dishes and lysed with RIPA lysis buffer (Solarbio, Beijing, China). Protein lysates were stored at −80°C until required for use. The CRISPR/Cas9‐mediated ITGAV knockout study was performed using KYSE30 and KYSE510 cells. HEK‐293T cells were co‐transfected with LentiCRISPRv2 plasmids and packaging plasmids (pMD2.G and psPAX2). After incubation for 48 h, lentivirus‐enriched medium was harvested and filtered. The filtered medium was supplemented with polybrene (8 µg/ml) and then applied to the target cells for 12–16 h. The infected cells were selected in complete growth medium supplemented with 4 µg/ml of puromycin (Sigma‐Aldrich) for 24–48 h before use in subsequent in vitro experiments.

### 2.5 Western blotting

Cells were harvested via scraping and washed twice with pre‐cooled PBS. The cells were lysed with RIPA buffer on ice for 1 h. Tumour tissues were cut into small pieces and homogenized using a taper‐type homogenizer. After centrifugation at 13000 rpm for 25 min, the protein concentration was quantified using a BCA Quantification Kit (Solarbio). Fifty micrograms of extracted protein was boiled with loading buffer at 98°C for 8 min and then separated by SDS‐PAGE. Separated proteins were then transferred to polyvinylidene difluoride membranes. After blocking with 5% BSA (diluted in PBST) for 90 min at room temperature, each membrane was then incubated with one of the following antibodies (1:1000): ITGAV, ITGA2, ITGA3, ITGA4, ITGA6, ITGA11, ITGB1, ITGB3, ITGB5, ITGB6, ITGB8, SYVN1, p‐FAK (Tyr 925), FAK, p‐PI3K p85α (Tyr 467), PI3K p85α, p‐AKT (Ser 473), AKT, p‐GSK3β (S9), GSK3β, cyclin D1, CDK4, CDK6, p‐SMAD2, p‐SMAD3 or β‐actin with gentle shaking at 4°C overnight. The next day, the membranes were washed five times with PBST before incubation with the appropriate secondary antibody at room temperature for 2 h. The membranes were then washed an additional five times with PBST before band detection by chemiluminescence (GE Healthcare, Little Chalfont, UK).

### 2.6 MTT assay

Cells were seeded into a 96‐well plate at a density of 2000 cells/well and then incubated at 37°C overnight. Various concentrations of each drug (indomethacin, cilengitide and iRGD) were diluted in medium, added to the 96‐well plates, and incubated for between 24 and 96 h at 37°C. MTT (Solarbio) powder was dissolved in sterilized PBS at a concentration of 5 mg/ml and filtered. Twenty microlitres of MTT was added to plates, followed by 1 h incubation at 37°C. The medium was then aspirated from the plates and replaced with 100 µl of DMSO. The plates were then gently agitated before the absorbance of each well was determined using a microplate reader.

### 2.7 Soft‐agar colony formation assay

To determine the anchorage‐independent cell growth using the soft‐agar colony formation assay, normal growth medium containing 0.5% agar was added to each well of a 6‐well plate and allowed to solidify at room temperature for 2 h. ESCC cells suspended at 8000 cells/well in normal growth medium containing 0.33% agar at the indicated concentrations of indomethacin were subsequently plated over the solidified bottom layer. The plates were then incubated at 37°C for 3 weeks. Afterwards, the colonies were photographed using an inverted microscope and quantified using the Image‐Pro Plus software.

### 2.8 Migration and invasion assays

Migration and invasion assays were performed using 24‐well trans well plates (Corning, NY, USA). For the migration assay, ESCC cells were seeded in upper chamber at 1 × 10^5^/well in 80 µl of RPMI‐1640 supplemented with 0.1% FBS. For the invasion assay, the chambers were coated with Matrigel (Corning) before seeding the cells (1 × 10^6^/well). Four hundred microlitres of RPMI‐1640 supplemented with 20% FBS was added to the lower chamber of each well. After a 48‐h incubation period, the upper chambers were removed and washed three times with PBS. The adherent cells were then fixed with 100% methanol for 15 min. Next, the cells were stained with 1% crystal violet (25% methanol) for 20 min and washed three times with PBS. The inner surface of the upper chamber was gently cleaned with a cotton swab. The upper chamber membranes were photographed using an inverted microscope and the stained cells were quantified using the Image‐Pro Plus software.

### 2.9 Cell cycle analysis

ESCC cells were seeded into 6‐cm dishes at a density of 2 × 10^5^ cells/dish in RPMI‐1640 without FBS. After incubation for 24 h, the cells were then treated with DMSO or indomethacin in RPMI‐1640 supplemented with 10% heat‐inactivated FBS. After 48 h, the cells were trypsinized, washed twice with ice‐cold PBS, fixed in 1.5 ml of 70% ice‐cold ethanol, and stored at −20°C for 12 h. After rehydration, the cells were digested with 100 mg/ml of RNase and stained with 20 mg/ml of propidium iodide (PI). Fluorescence measurements were obtained using a flow cytometer (BD, San Diego, CA, USA).

### 2.10 Annexin V apoptosis assay

KYSE30 and KYSE510 ESCC cells were plated into 6‐cm plates at a density of 1 × 10^5^/dish. After incubation for 24 h, various concentrations of indomethacin (dissolved in RPMI‐1640 medium supplemented with 10% heat‐inactivated FBS) were added to each plate for 72 h. Afterwards, the cells were harvested, washed three times with ice‐cold PBS, and subjected to staining with annexin V and PI. Fluorescence measurements were obtained using a flow cytometer (BD).

### 2.11 Pulldown assay

ESCC cell lysates (500 µg) or ITGAV recombinant proteins (200 ng) were mixed with 50 µl of indomethacin, disulfiram‐Sepharose 4B, or DMSO‐Sepharose 4B beads in reaction buffer (2 mg/ml BSA, 50 mM Tris‐HCl pH 7.4, 200 mM NaCl, 5 mM EDTA, 1 mM dithiothreitol and 0.01% NP40). The mixtures were then rotated at 4°C for 12 h. Afterwards, the beads were washed five times with washing buffer (200 mM NaCl, 50 mM Tris pH 7.4, 5 mM EDTA, 0.01% NP40 and 1 mM dithiothreitol) at 4°C. After several washes, the proteins were eluted from the beads through boiling in loading buffer at 95°C for 5 min. Protein binding was then assessed using Western blotting.

### 2.12 *In silico* molecular docking

*In silico* molecular docking was performed using the Schrödinger Suite 2016 software. To model the binding of indomethacin with ITGAV, the crystal structure of ITGAV was downloaded from the PDB database (www.rcsb.org/pdb [PDB] code: 4o02) and prepared under the standard procedures of the Protein Preparation Wizard (Schrödinger Suite 2016). All water molecules in the structure were removed and hydrogen atoms were added to the protein prior to docking. The LigPrep program was used to prepare the indomethacin for the docking study. Docking was performed with the Glide program under extra precision mode using the default parameters.

### 2.13 Surface plasmon resonance

Surface plasmon resonance (SPR) analysis using the Biacore T‐200 (GE Healthcare, Waukesha, WI, USA) was conducted in order to evaluate the interaction between indomethacin and ITGAV. ITGAV protein was covalently immobilized at densities of 200 response units onto a CM5 sensor chip. Next, indomethacin (< 1% (v/v) DMSO) was injected at concentrations ranging between 15.625 and 500 µM at 25°C. The change in the refractive index due to the drug–protein interaction was measured in real time, which allowed for plotting of the interaction results as response units versus time. The interaction results were analysed using the BIA evaluation 3.0 software.

### 2.14 Real‐time PCR

KYSE30 and KYSE510 cells were seeded into 10‐cm dishes and treated with DMSO or indomethacin for 24 h. Cells were washed with cold PBS and transferred into 1.5 ml tubes. TRIzol was used according to the manufacturer's instructions to extract total RNA. cDNA was synthesized using the FastQuant RT Kit (Tiangen Biotech, Beijing, China), and quantitative Real‐time PCR (RT‐PCR) was performed using the SYBR® Premix Ex Taq™ Kit (TAKARA, Dalian, China). The primers used in the PCR reactions are as follows: human ITGAV: 5′ ATCTGTGAGGTCGAAACAGGA‐3′ and 5′TGGAGCATACTCAACAGTCTTTG‐3′; human GAPDH: 5′‐CAAGGTCATCCATGACAACTTTG‐3′ and 5′‐GTCCACCACCCTGTTGCTGTAG‐3′.

### 2.15 Co‐immunoprecipitation

Cells were harvested and lysed with RIPA buffer (Solarbio) at 4°C. The cell lysates were mixed with indicated antibody at 4°C overnight. The next day, protein A/G plus agarose beads (Santa Cruz Biotechnology) were added; the mixture was then rotated for 2 h at 4°C. Next, the beads were washed four times with RIPA buffer and boiled for 7 min in loading buffer. The eluted protein was subjected to Western blotting.

### 2.16 Immunofluorescence

Seeded cells were allowed to adhere onto glass coverslips in 24‐well plates at 37°C for 12 h. Cells were treated with DMSO or various concentrations of indomethacin for 24 h. The cells were then washed three times with ice‐cold PBS and fixed with 100% methanol for 15 min. After fixation, the cells were washed three times with PBS and incubated at 4°C overnight with ITGAV antibody in PBS containing 3% BSA, 0.3% Triton X‐100 and 10% goat serum. The next day, slides were washed three times and incubated with secondary antibody in PBS containing 3% BSA for 1 h at 24°C. After several washes, slides were counterstained with DAPI (Solarbio). The coverslips were mounted on microscope slides and imaged using confocal microscopy (Nikon, Tokyo, Japan).

### 2.17 Cytokine ELISA assays

Blood was collected from mice and the serum was harvested by centrifugation at 3500 rpm for 10 min at 4°C. A human TGFβ kit (Abcam) was used for cytokine measurements. Tumour tissues were harvested, cut into small pieces, and homogenized in tissue lysis buffer (Solarbio). Proteins were extracted by centrifugation at 13500 rpm for 15 min 4°C. Protein concentration was quantified using a BCA kit (Solarbio). IFNγ was measured using an ELISA kit (Liankebio, Hangzhou, China), according to the manufacturer's protocol.

### 2.18 Flow cytometry

Mice blood was harvested in heparin lithium‐anticoagulant tubes. Red blood cells were then lysed with red blood cell lysis solution (Solarbio) at room temperature for 30 min. Next, the intact cells were washed two times with PBS and blocked with FcR‐blocking reagent (Miltenyi, Beijing China) for 15 min. The cells were then stained with following antibodies: anti‐human CD45‐FITC antibody (Biolegend, San Diego, USA), anti‐human CD3‐FITC (BD Pharmingen), anti‐human CD4‐PE (Biolegend), and anti‐human CD8‐PerCP‐Cy5.5 (BD Pharmingen) for 30 min at 4°C. Cell fractions were then measured using a BD flow cytometer. Data were analysed with the FlowJo software.

### 2.19 Mouse xenograft models

Mice were housed under specific pathogen‐free conditions. All animal experiments were approved by the Bioethics Committee of Zhengzhou University and followed guidelines set by the Institutional Animal Care and Use Committee (CUHCI2019002, CUHCI2021001 and CUHCI2021005). To generate the cell‐derived xenograft mouse model, *nu/nu* nude mice (Vital River Labs., Beijing, China) were subcutaneously injected with 1 × 10^7^ cells (KYSE30 and KYSE510). Mice were randomly divided into three groups: (1) shMock, (2) shITGAV#1 and (3) shITGAV#5. For the patient‐derived xenograft (PDX) mouse model, 6‐ to 8‐week‐old non‐obese diabetic/severe combined immunodeficient (NOD‐SCID) female mice (Vital River Labs., Beijing, China) were used for the animal experiments. Tumour tissue fragments were collected from ESCC patients and implanted subcutaneously into the mice. The tumours were passaged for three additional generations; P3–P6 tumours were used for subsequent studies. Once tumour volumes reached approximately 200 mm^3^, the mice were divided into three treatment groups as follows: (1) control group, (2) 1 mg/kg indomethacin and (3) 4 mg/kg indomethacin. Indomethacin or vehicle (5% DMSO in PBS) was given by intra‐gastric administration every day. For knockdown experiments, concentrated lentivirus was injected at multiple tumour sites. The mice were divided into three groups as follows: (1) shMock, (2) shITGAV#1 and (3) shITGAV#5. Tumour volume was calculated as follows: length × width × height × 0.5. The in vivo studies were terminated once the tumour volume reached approximately 1 cm^3^, after which the mice were euthanized, and the tumours were extracted. For the recurrence mouse model, tumour tissue fragments were inoculated subcutaneously in mice. The tumours were removed, once their volumes reached approximately 500 mm^3^. Mice were divided randomly into four groups (*n* = 8): (1) control group, (2) 4 mg/kg indomethacin, (3) 100 mg/kg iRGD and (4) 100 mg/kg cilengitide. Indomethacin was given by intra‐gastric administration and integrin inhibitors (iRGD and cilengitide) were administered by intra‐peritoneal (IP) injection every day over the course of 43 days. Tumour recurrence was defined as a 50 mm^3^ growth observed at location that the original tumour was excised from. To determine the anti‐tumour effect of indomethacin on immune‐competent mice, we generated a humanized mouse model.^35^, ^36^ BRGSF mice were inoculated with PDX tumours and then divided into four groups: (1) control group, (2) PBMC group, (3) 4 mg/kg indomethacin and (4) PBMC plus 4 mg/kg indomethacin. Indomethacin was administered for 18 days. Human PBMCs were injected into mice tail veins 1 week after ESCC tumour inoculation. The concentration of human CD45 present within the blood of the mice was measured on days 13 and 23. The blood and tumour from the mice within this study were harvested and used for in vitro assays.

### 2.20 Statistics

SPSS software (Armonk, NY, USA) and Graph Pad Prism (San Diego, CA, USA) were used for statistical analysis. Data are expressed as the mean value ± SD from at least three independent experiments. Statistical significance was calculated by two‐tailed Student's *t* test, chi‐square test, one‐way ANOVA, or two‐way ANOVA. Statistical significance is indicated by ‘*, *p *< 0.05; **, *p* < 0.01; ***, *p* < 0.001’.

## 3 RESULTS

### 3.1 ITGAV is upregulated in ESCC and associated with poor prognosis

To evaluate the mRNA expression of integrin family members (ITGA1‐11, ITGAV and ITGB1‐8) in the context of ESCC, expression profiles detailing the abundance of integrin transcripts within normal oesophageal and ESCC tumour tissues were downloaded from The Cancer Genome Atlas (TCGA) database (http://ualcan.path.uab.edu) and analysed^37^ (Figure S1A). Results illustrated that ITGA2, ITGA3, ITGA4, ITGA6, ITGA11, and ITGAV transcripts were increased in ESCC tumour tissues (*n* = 95) compared with normal tissues (*n* = 11). However, ITGA8 transcript was decreased in tumour tissues compared with normal tissues. No significant differences in mRNA expression levels were observed for other integrin α‐subunits between normal and tumour tissues. Among the integrin β‐subunits, ITGB1, ITGB2, ITGB4, ITGB5, ITGB6, ITGB7, and ITGB8 were highly expressed in tumour tissues compared with normal tissues. In contrast, there was no significant difference in ITGB3 mRNA expression levels between the two groups (Figure S1A).

**FIGURE 1:**
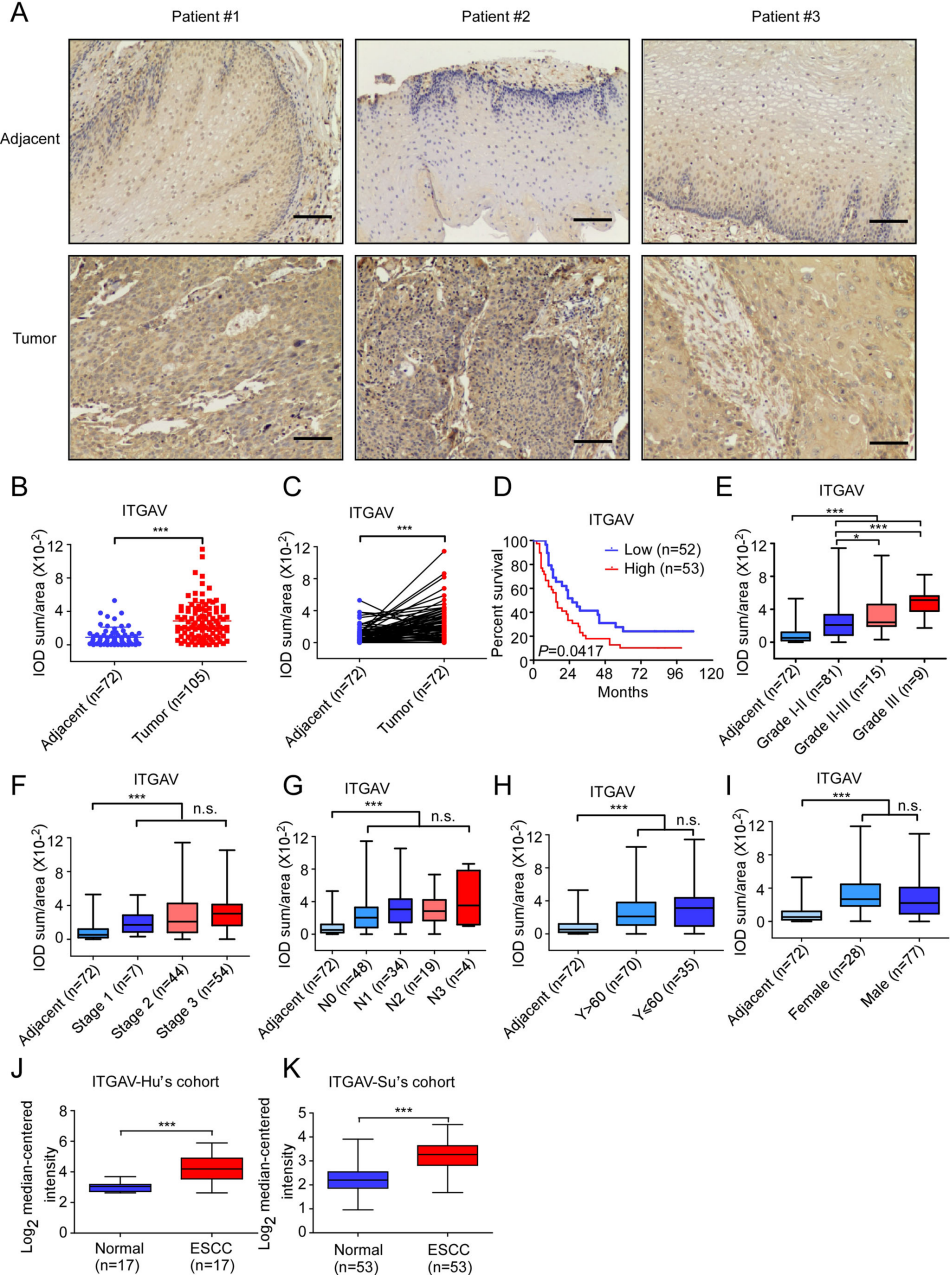
ITGAV is highly upregulated in ESCC. (A) Representative images showing ITGAV expression in clinical tissues (100× magnification). Scale bar: 50 µm. (B) ITGAV protein levels in ESCC and adjacent tissue samples were assessed by IHC. The integrated optical density (IOD)/area × 10^−2^ was calculated using Image‐Pro Plus 6.0 software. (C) Quantification of ITGAV expression in paired samples. (D) Kaplan–Meier analysis of the overall survival of 105 patients with ESCC, stratified according to ITGAV protein level (high or low). (E–I) ITGAV protein levels in the tissues of ESCC patients grouped by tumour grade, tumour stage, lymph node metastasis, age, and sex. (J and K) ITGAV mRNA levels were compared between ESCC tissues and normal tissues in multiple patient cohorts. The original data were retrieved from the Oncomine database and plotted. *: *p* < 0.05; ***: *p* < 0.001. Data are presented as the mean value ± SD (error bar)

We next verified the protein levels of the integrin α‐subunits suggested to be differentially represented between samples at the mRNA level, including ITGA2, ITGA3, ITGA4, ITGA6, ITGA11, and ITGAV using 10 paired (tumour and adjacent) ESCC patient tissues. Results showed that ITGAV (10/10), ITGA2 (7/10), ITGA3 (8/9), ITGA4 (7/10), ITGA6 (7/8), and ITGA11 (7/9) protein levels were increased in tumour tissues compared with adjacent tissues (Figure S1B). Among the integrin α‐subunits that were examined, ITGAV showed increased expression in all 10 paired ESCC tissues. We next performed IHC staining using a tissue array, including tumour tissues (*n* = 105) and adjacent tissues (*n* = 72), to determine the protein levels of ITGAV. Individual patient information is provided in the Supplementary File. The results of the IHC analysis showed that ITGAV was upregulated in ESCC tumour tissues compared to adjacent tissues (Figure 1B and C). Moreover, we also observed ITGAV expression in stroma cells (Figure 1A). Representative images illustrating ITGAV expression in paired tissues are shown in Figure 1A. Increased ITGAV expression was associated with a poor survival outcome and clinical grade (Figure 1D and E; Table 1). However, there were no significant differences observed in ITGAV protein levels based on clinical stage, lymph node metastasis, age, or gender (Figure 1F–I). Oncomine dataset analysis (https://www.oncomine.org) indicated that ITGAV expression was significantly upregulated in ESCC tissues compared to normal tissues (Figure 1J and K). The protein levels of the ITGAV β‐subunits in tumour and adjacent tissues were determined by Western blotting (Figure S1B). The results showed that ITGB1 (4/10), ITGB3 (6/10), ITGB5 (6/10), and ITGB6 (5/10) protein levels were increased in tumour tissues compared to adjacent tissues. In contrast, ITGB8 (10/10) protein levels were increased in adjacent tissues compared with tumour tissues. These results indicated that integrin αvβ3 is upregulated in ESCC and that ITGAV expression is positively correlated with poor survival rates in ESCC patients.

**TABLE 1:** Cohort characteristics of ESCC patient

Characteristics	ITGAV expression levels		
	Low (*n* = 52)	High (*n* = 53)	*p* ^b^
Gender			
Male	40 (76.9%)	37 (71.2%)	0.41
Female	12 (23.1%)	16 (28.8%)	
Age			
≤60	15 (28.8%)	20 (38.5%)	0.33
>60	37 (71.2%)	33 (61.5%)	
Pathology grade			
I–II	45 (86.5%)	36 (69.2%)	0.0297
II–III	6 (11.5%)	9 (17.3%)	
III	1 (1.9%)	8 (15.4%)	
T classification^a^			0.0395
T1	5 (9.6%)	0 (0.0%)	
T2	5 (9.6%)	7 (13.5%)	
T3	42 (80.8%)	43 (82.7%)	
T4	0 (0.0%)	3 (5.8%)	
N classification			0.217
N0	29 (55.8%)	19 (36.5%)	
N1	14 (26.9%)	20 (38.5%)	
N2	7 (13.5%)	12 (23.1%)	
N3	2 (3.8%)	2 (3.8%)	
Clinical stage			0.139
1	5 (9.6%)	2 (3.8%)	
2	25 (48.1%)	19 (36.5%)	
3	22 (42.3%)	32 (61.5%)	
4	0 (0.0%)	0 (0.0%)	

^a^ Tumour TNM staging components, including Tumour (T), Node (N) and Metastasis (M), were defined according to the American Joint Committee on Cancer (AJCC) 7th edition.

^b^ Chi‐square text.

### 3.2 ITGAV promotes ESCC cell proliferation and metastasis

**FIGURE 2:**
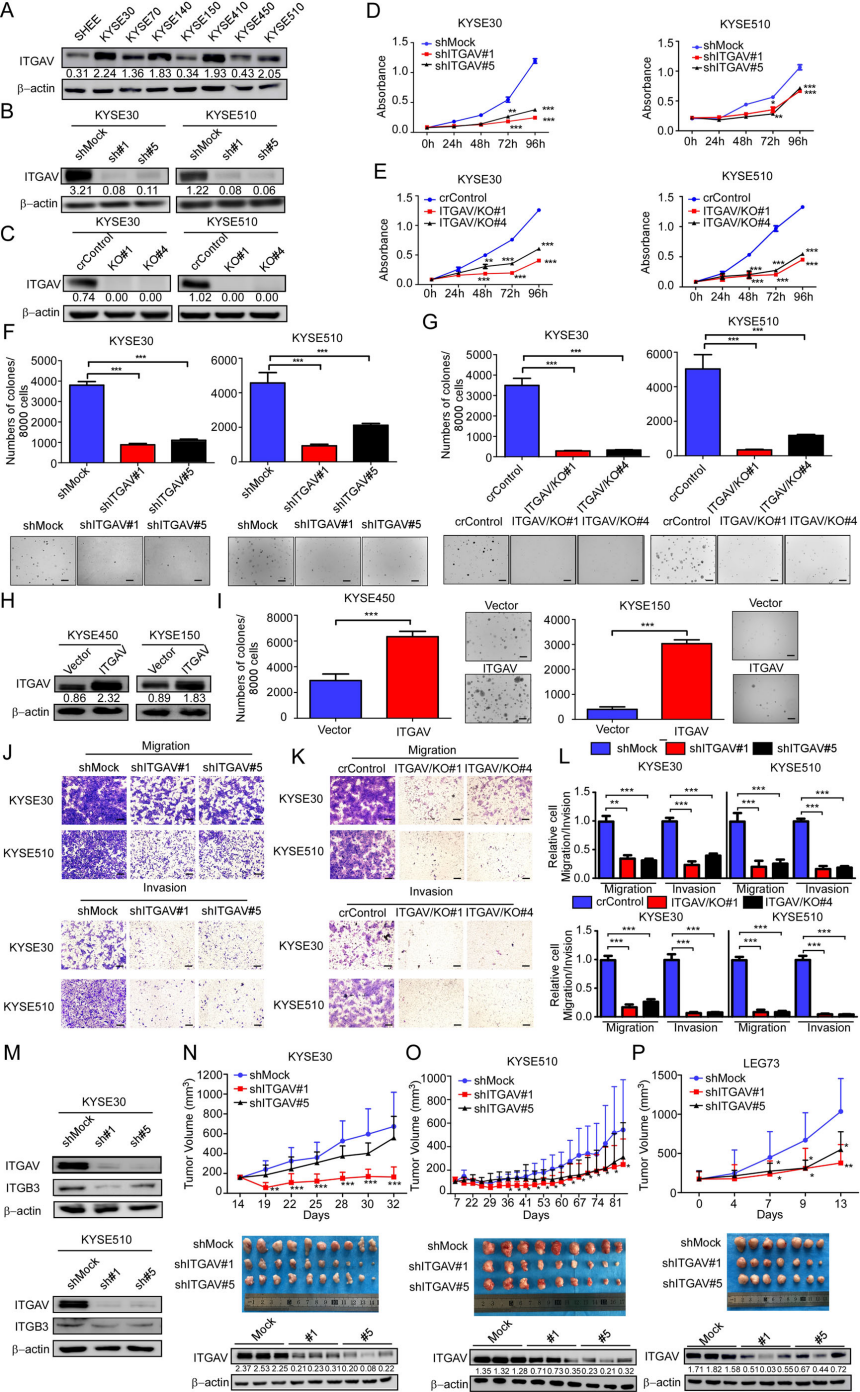
Silencing of ITGAV inhibits ESCC cell proliferation and tumour growth. (A) ITGAV protein levels in immortalized SHEE oesophageal epithelial cells and ESCC cells were detected by Western blotting. (B and C) Silencing efficiencies of ITGAV constructs were determined by Western blotting. Cell proliferation was evaluated by MTT (D and E) and anchorage‐independent cell growth (F and G) assays after ITGAV knockdown or knockout in KYSE30 and KYSE510 cells. Scale bar: 400 µm. (H) ITGAV protein levels were detected after the overexpression of ITGAV in KYSE450 and KYSE150 cells. (I) The anchorage‐independent cell growth assay was performed using vector‐ and ITGAV‐transfected cells. Scale bar: 400 µm. (J–L) Cell migration and invasion were determined by Transwell migration and invasion assays. Scale bar: 400 µm. (M) ITGAV and ITGB3 protein levels were determined after ITGAV knockdown. (N and O) *Nu/nu* mice were injected with shMock and shITGAV ESCC cells; the size of the resulting tumours was monitored twice per week. KYSE30: *n* = 10/group; KYSE510: *n* = 9/group. (P) LEG73 tumour fragments were inoculated into NOD‐SCID mice (*n* = 7/group). Lentivirus was injected at multiple points into the tumours. Tumour size was monitored twice per week. For (L–N), the tumours were photographed, and the ITGAV protein levels in tumours were determined at the end of the experiment. *: *p* < 0.05; **: *p* < 0.01; ***: *p* < 0.001. For (A–M), three independent experiments were performed. Data are presented as the mean value ± SD (error bar)

The ITGAV protein levels in SHEE and ESCC cells (KYSE30, KYSE70, KYSE140, KYSE410 and KYSE510) were examined by Western blotting. Compared to SHEE cells, KYSE30, KYSE140, KYSE410, and KYSE510 cells showed increased ITGAV protein levels (Figure 2A). As ITGAV is frequently upregulated in ESCC, we hypothesized that the downregulation of ITGAV may inhibit ESCC cell proliferation. Indeed, after knockdown or knockout of ITGAV, the cell growth was suppressed (Figure 2B–G). The downstream effectors of ITGAV, including p‐FAK, p‐PI3K, and p‐AKT, were also downregulated after knockdown of ITGAV (Figure S2A). In contrast, the overexpression of ITGAV promoted the colony formation ability in ESCC cells (Figure 2H and I) and activated the FAK/PI3K/AKT signalling pathway (Figure S2B). Figure 2J–L illustrates that downregulation of ITGAV inhibited cell migration and invasion. Furthermore, we detected the protein level of the integrin β‐subunits (ITGB1, ITGB3, ITGB5, ITGB6 and ITGB8) after knockdown of ITGAV. The results showed that only ITGB3 expression was significantly decreased in the KYSE30 and KYSE510 knockdown groups, while ITGB1 expression was decreased in the KYSE30 knockdown group (Figure 2M and Figure S2C). To further investigate the function of ITGAV in vivo, cell line‐derived xenograft models were generated through injecting athymic nude mice with shMock or shITGAV cells. The results showed that ITGAV knockdown suppressed tumour growth in the shITGAV KYSE30 or shITGAV KYSE510 groups (Figure 2N and O, Figure S2E and F). Moreover, the ITGAV protein levels in the shITGAV groups were reduced (Figure 2N and O). Although cell line‐derived xenografts are routinely used to assess tumour dynamics in vivo, they may not accurately recapitulate the tumour behaviour observed in patients. Therefore, to investigate the potential function of ITGAV in ESCC patient‐derived tumours, a lentivirus‐transduced PDX mouse model was generated. The data suggested a significant reduction in the volume and weight of shITGAV‐infected PDX tumours (Figure 2P and Figure S2G). These data suggest that ITGAV expression contributes to ESCC cell proliferation and tumour growth.

### 3.3 Indomethacin binds to ITGAV and modulates its downstream signalling effectors

**FIGURE 3:**
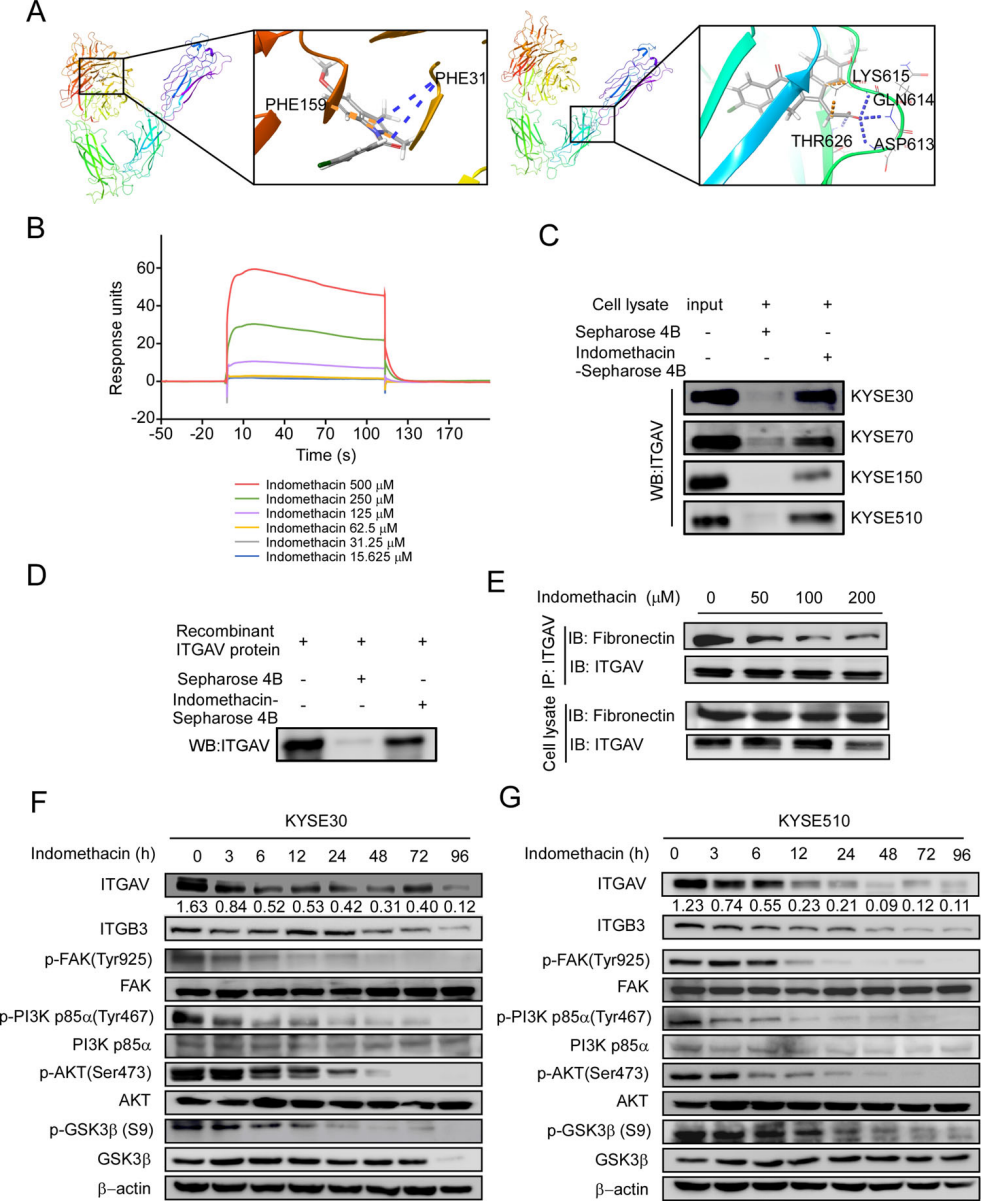
Indomethacin binds to ITGAV. (A) Computational modelling illustrating binding between indomethacin and ITGAV. Indomethacin binds to ITGAV at PHE31 and PHE159 (*upper panel*) or LYS615, GLN614, and ASP613 (*lower panel*). The ITGAV structure is visualized as a ribbon; indomethacin is visualized as a stick. (B) Binding sensorgrams for the interaction between indomethacin and immobilized ITGAV. The ITGAV immobilization level was 200 RU. ESCC cell lysates (C) or ITGAV recombinant proteins (D) were mixed with 50 µl of indomethacin‐conjugated Sepharose 4B beads or DMSO‐conjugated Sepharose 4B beads in reaction buffer. The pulled‐down proteins were detected by Western blotting. (E) ITGAV‐overexpressing HEK‐293T cells were lysed and added to 20 µg of fibronectin proteins. After incubation with the indicated concentrations of indomethacin at 4°C overnight, proteins were subjected to IP assay. (F and G) ITGAV, ITGB3, p‐FAK, FAK, p‐PI3K, PI3K, p‐AKT, AKT, p‐GSK3β, and GSK3β protein levels were assessed in KYSE30 and KYSE510 cells by Western blotting after treatment with DMSO or 200 µM indomethacin (0–96 h). For (B–G), three independent experiments were performed

In order to identify a new ITGAV inhibitor that can suppress ESCC growth and prevent recurrence, we performed a virtual screening of FDA‐approved drugs. The top 20 drugs that showed significant docking scores (−7.2 to −4.7 kcal/mol) are listed in Table S1, including pemetrexed acid, oxaliplatin, gemcitabine, lapatinib, and sorafenib. In this study, we aimed to identify an affordable, minimally toxic FDA‐approved drug that was not originally purposed as a cancer chemopreventive agent. Table S1 illustrates that indomethacin (docking score: −5.30) and disulfiram (anti‐alcoholic drug) (docking score: −4.93) are promising candidates. We next used a docking model to verify the results of the virtual screening. The docking results suggested that indomethacin can bind to ITGAV at PHE31 and PHE159 or LYS615, GLN614, and ASP613 (Figure 3A). Additionally, we performed SPR analysis to explore the binding affinity of indomethacin to ITGAV. Serial concentrations of indomethacin were perfused onto immobilized ITGAV (Figure 3B). The results indicated that indomethacin had a dose‐dependent affinity for ITGAV. To further confirm this interaction, in vitro pulldown assays with indomethacin‐conjugated Sepharose 4B beads were performed using recombinant ITGAV protein or KYSE30, KYSE70, KYSE150, and KYSE510 cell lysates. The results illustrated that the indomethacin‐conjugated beads were able to bind to ITGVA protein (Figure 3C and D). The pulldown assay was also performed using disulfiram‐conjugated Sepharose 4B beads; however, the results showed that disulfiram could not bind to ITGAV protein (Figure S3A). We next determined whether indomethacin could bind to the β‐subunits of ITGAV. The results indicated that indomethacin could not bind to ITGB1, ITGB3, ITGB5, ITGB6, or ITGB8 (Figure S3B). To further assess whether indomethacin can compete with the ITGAV for its interaction with fibronectin, we performed a competition IP assay using HEK‐293T cells overexpressing ITGAV. The result suggested that indomethacin decreases the affinity between ITGAV and fibronectin in a concentration‐dependent manner (Figure 3E). To determine the effect of indomethacin on the downstream protein targets of ITGAV, we treated ESCC cell lines with indomethacin and quantified changes in protein levels using Western blotting. The results showed that KYSE30 and KYSE510 cells treated with 200 µM indomethacin exhibited decreased ITGAV, ITGB3, p‐FAK (Tyr925), p‐PI3K p85a (Tyr467), p‐AKT (Ser473), and p‐GSK3β (S9) protein levels (Figure 3F and G). These findings indicate that indomethacin may disrupt the interaction between ITGAV and fibronectin and also decreased integrin αvβ3 protein levels, thereby attenuating the downstream effectors of integrin αvβ3.

### 3.4 Indomethacin promotes SYVN1‐mediated degradation of ITGAV

**FIGURE 4:**
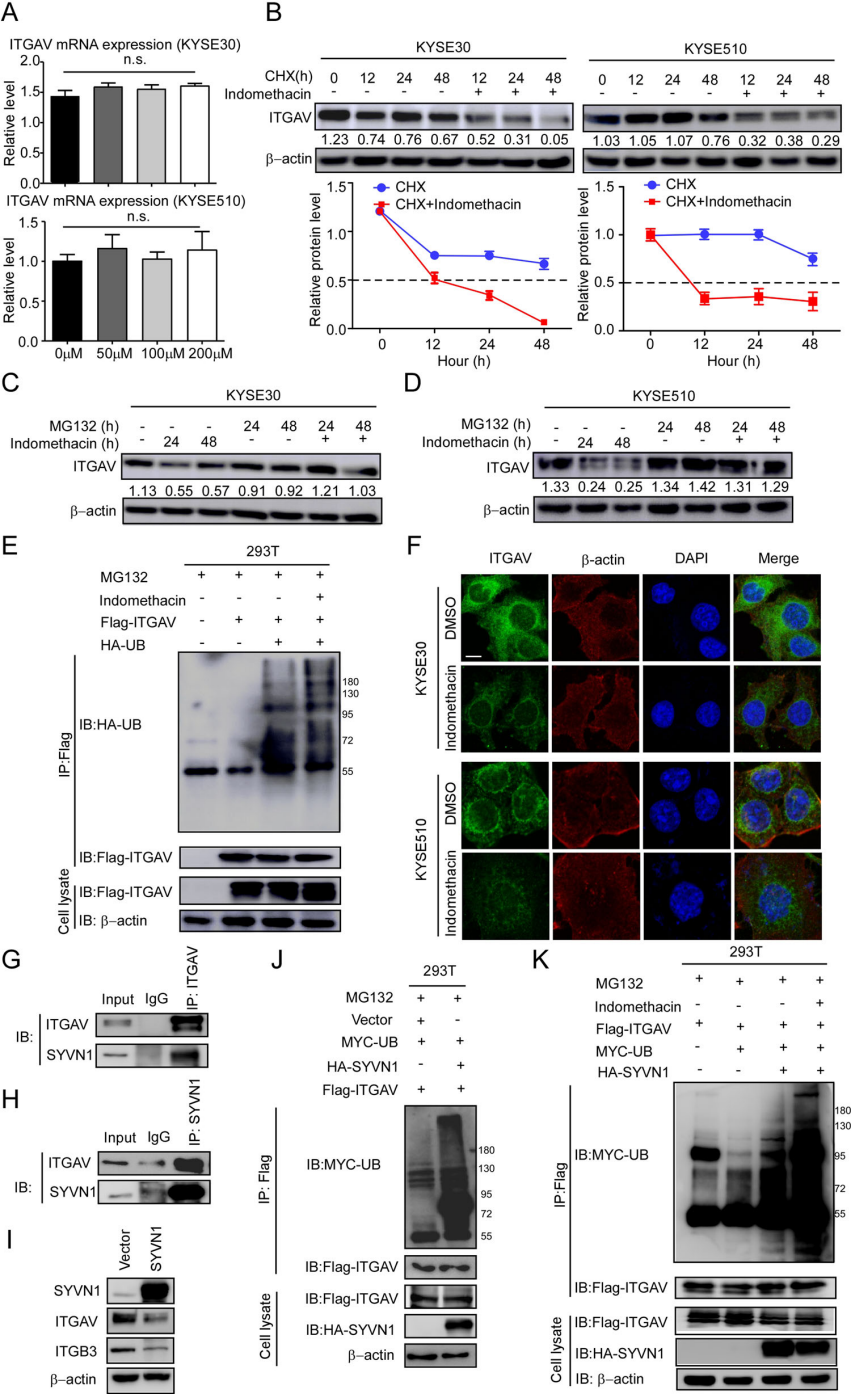
Indomethacin promotes ITGAV degradation through the ubiquitin‐proteasome pathway. (A) ITGAV mRNA expression was detected by RT‐PCR. Gene expression was expressed as a fold change value relative to that in KYSE30 and KYSE510 cells treated with 200 µM indomethacin for 24 h. (B) Time course of ITGAV degradation. CHX (100 µg/ml) was added to ESCC cells, and the cells were treated with DMSO or 200 µM indomethacin for the indicated times. The ITGAV band intensities were quantified by ImageJ software. The dotted line (—) indicates the half‐life (T½) of ITGAV protein in cells. (C and D) ESCC cells (KYSE30 and KYSE510) were incubated with MG132 and then treated with 200 µM indomethacin or DMSO for the indicated times. ITGAV protein level was assessed by Western blotting. (E) HEK‐293T cells were co‐transfected with flag‐ITGAV and HA‐ubiquitin plasmids, pre‐treated with MG132 for 12 h and then incubated with indomethacin (200 µM) for 2 days. The cells were harvested and lysed in lysis buffer, followed by immunoprecipitation with an anti‐flag antibody. The ubiquitinated ITGAV was determined by Western blotting. (F) KYSE30 and KYSE510 cells were treated with DMSO or indomethacin for 24 h, followed by the detection of ITGAV protein levels using immunofluorescence. Scale bar: 10 µm. DAPI and β‐actin were used as internal controls. (G) KYSE30 cells were lysed and processed for an IP assay with IgG or anti‐ITGAV antibodies. Interactions between ITGAV and SYVN1 were detected by Western blotting. (H) KYSE30 cells were lysed and processed for an IP assay with IgG or anti‐SYVN1 antibodies. Interactions between ITGAV and SYVN1 were detected by Western blotting. (I) KYSE30 cells were transfected with HA‐vector or HA‐SYVN1 plasmids for 48 h. Cells were harvested and lysed. Proteins were visualized by Western blotting. SYVN1, ITGAV and ITGB3 protein levels are shown in the vector group and SYVN1‐overexpressing group. (J) HEK‐293T cells were co‐transfected with MYC‐UB, HA‐SYVN1 and Flag‐ITGAV plasmids. Cells were treated with MG132 (10 µM) for 12 h before harvesting. Cells were processed for an IP assay with anti‐flag antibody. Ubiquitinated ITGAV was detected by Western blotting. (K) HEK‐293T cells were co‐transfected with Flag‐ITGAV, MYC‐UB and HA‐SYVN1, pre‐treated with MG132 (10 µM) for 12 h and then incubated with indomethacin (200 µM) for 2 days. The cells were harvested and lysed in lysis buffer. Lysates were then processed for an IP assay with an anti‐flag antibody. Ubiquitinated ITGAV was determined by Western blotting. Three independent experiments were performed. Data are presented as the mean value ± SD (error bar)

ITGAV protein levels were downregulated after indomethacin treatment. Therefore, we investigated whether indomethacin can decrease ITGAV mRNA transcription. We examined ITGAV mRNA levels by RT‐PCR. The total RNA from indomethacin‐treated ESCC cells was extracted and subjected to RT‐PCR. The results showed that indomethacin treatment did not decrease the ITGAV mRNA levels (Figure 4A), indicating that a mechanism occurring post‐translation may account for decreased protein levels. To explore this possibility, we used cycloheximide (CHX), a protein synthesis inhibitor, to investigate the effect of indomethacin on ITGAV protein stability. The results showed that the half‐life of ITGAV protein in the indomethacin plus CHX treatment group was shorter than that of the CHX treatment group (Figure 4B), suggesting that indomethacin promotes ITGAV degradation in KYSE30 and KYSE510 cells. We next sought to determine whether ITGAV was degraded via the ubiquitin proteasome pathway. ESCC cells were treated with indomethacin plus MG132, a proteasome inhibitor, and then ITGAV protein levels were assessed by Western blotting. The data showed that MG132 successfully attenuated the indomethacin‐mediated degradation of ITGAV (Figure 4C and D). The ubiquitination activity assay showed that more ubiquitin bound to ITGAV in indomethacin‐treated cells than in DMSO‐treated cells, indicating that ITGAV was ubiquitinated during the indomethacin‐induced proteasomal degradation (Figure 4E). Immunofluorescence staining also showed that the ITGAV degradation was increased in the indomethacin treatment groups (Figure 4F). As E3 ubiquitin ligases play important roles in the ubiquitin proteasome system, we next determined whether indomethacin treatment enhanced E3 ubiquitin ligase‐mediated ubiquitination of ITGAV. We used Ubibrowser (http://ubibrowser.ncpsb.org.cn/ ubibrowser/) to predict the E3 ubiquitin ligases that could potentially interact with ITGAV (Figure S4A). Based upon the predictions made by Ubibrowser, we performed an IP assay with an anti‐ITGAV antibody to detect if ITGAV interacts with the top three (NEDD4, ITCH and SYVN1) predicted candidates. Additionally, we assessed whether two known E3 ubiquitin ligases (CBLC and CBL) of other integrin members could also promote ITGAV degradation^38^, ^39^ (Figure S4B). The results showed that the E3 ubiquitin ligase SYVN1 could bind to ITGAV (Figure 4G and H) in KYSE30 cells. We also observed that SYVN1 overexpression reduced integrin αvβ3 protein levels (Figure 4I). To further verify whether SYVN1 could induce ITGAV ubiquitination, we overexpressed ITGAV and SYVN1 in HEK‐293T cells and then detected the ubiquitination of ITGAV. Indeed, the results showed that ITGAV ubiquitination was increased in SYVN1 overexpressed cells (Figure 4J). These results suggest that SYVN1 binds to ITGAV and induces its ubiquitination. Additionally, indomethacin treatment enhanced SYVN1‐mediated ubiquitination of ITGAV (Figure 4K), presumably due to a conformational change induced by indomethacin. Overall, these results suggest that indomethacin binds to ITGAV and promotes SYVN1‐mediated ubiquitination of ITGAV.

### 3.5 Indomethacin inhibits ESCC cell growth by targeting ITGAV

**FIGURE 5:**
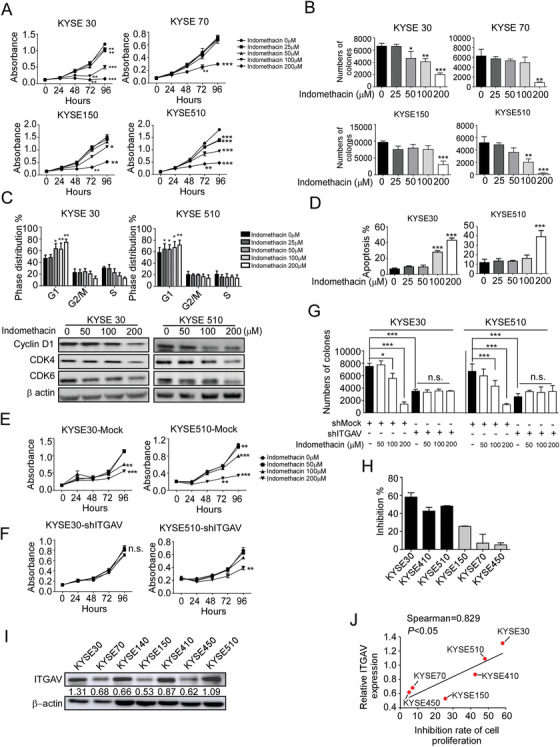
Indomethacin suppresses ESCC cell growth. (A) ESCC cells were treated with DMSO or different concentrations of indomethacin for various times (24, 48 or 96 h). Cell growth inhibition was detected by MTT assay. (B) Anchorage‐independent cell growth in ESCC cells treated with DMSO or various concentrations of indomethacin. (C) KYSE30 and KYSE510 cells were seeded onto 6‐cm dishes. After treatment with DMSO or various concentrations of indomethacin for 2 days, the cells were harvested for cell cycle analysis (upper panel). Cyclin D1, CDK4 and CDK6 protein levels were assessed by Western blotting (lower panel). (D) KYSE30 and KYSE510 cells were seeded in plates for 24 h and treated with DMSO or various concentrations of indomethacin. An apoptosis assay was performed after incubation for 72 h. (E–G) The effect of indomethacin treatment on ESCC cell growth and anchorage‐independent cell growth was assessed in KYSE30 and KYSE510 cells expressing shMock or shITGAV. *: *p* < 0.05; **: *p* < 0.01; ***: *p* < 0.001. (H) The inhibition rate of cell proliferation was determined in ESCC cell lines by MTT assay. (I) ITGAV protein levels in KYSE30, KYSE70, KYSE150, KYSE410, KYSE450 and KYSE510 ESCC cell lines were determined by Western blotting. (J) Correlation between the ITGAV protein levels and cell growth inhibition rate in various ESCC cell lines. Indomethacin was more effective in ESCC cells with increased ITGAV expression. The significance of the correlation was determined using the non‐parametric Spearman method. Four independent experiments were performed. Data are presented as the mean value ± SD (error bar)

To detect the inhibitory effect of indomethacin on ESCC cell proliferation, we treated ESCC cell lines with various concentrations of indomethacin at different time points and then measured cell viability using an MTT assay. We found that indomethacin could significantly inhibit ESCC cell growth (Figure 5A) without inhibiting normal oesophageal epithelial cell growth (Figure S5A). Additionally, indomethacin treatment strongly suppressed the colony formation ability of ESCC cells (Figure 5B). To determine whether indomethacin treatment affects cell cycle progression, cells were incubated with indomethacin for 48 h prior to being analysed using flow cytometry. The results indicated that indomethacin induced G1‐phase arrest (Figure 5C, upper panel, Figure S5B and C). We then examined the protein levels of G1‐phase‐associated proteins by Western blotting. The results showed that indomethacin treatment reduced cyclin D1, CDK4, and CDK6 protein levels in ESCC cells (Figure 5C, lower panel). Furthermore, to investigate whether indomethacin could induce apoptosis, annexin V staining was performed after indomethacin treatment for 72 h. We found that indomethacin treatment increased the rate of apoptotic cells (Figure 5D and Figure S5D). In order to determine whether the effects of indomethacin are dependent upon ITGAV expression, we performed MTT and anchorage‐independent cell growth assays in shMock or shITGAV cells after DMSO or indomethacin treatment (KYSE30 and KYSE510). The results showed that indomethacin treatment produced less of an inhibitory effect with respect to cell proliferation (Figure 5E and F) and anchorage‐independent colony growth (Figure 5G) in ITGAV knockdown ESCC cells compared to shMock cells. We then compared the rates of cell growth inhibition (Figure 5H) with the respective ITGAV protein levels in the infected cells (Figure 5I). Our results showed that cells with increased ITGAV protein levels were more sensitive to indomethacin treatment than cells with low ITGAV protein levels. This observation suggests that the ITGAV protein levels, but not COX1/2 protein levels, may be causally associated with the inhibitory activity of indomethacin (Figure 5J, Spearman = 0.829, *p* < 0.05) (Figure 5H–J and Figure S5F–H).

### 3.6 Indomethacin inhibits ESCC PDX tumour growth

**FIGURE 6:**
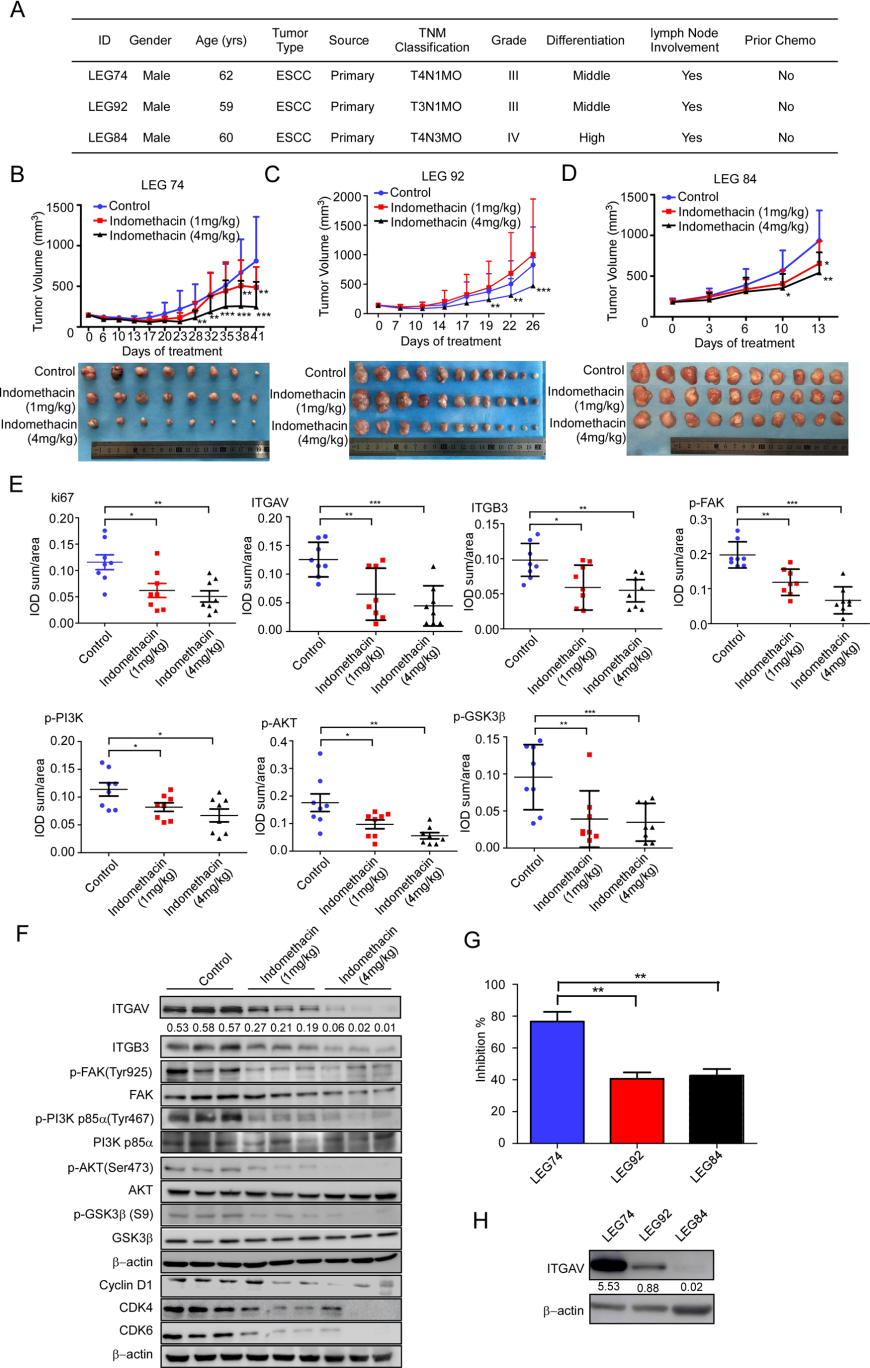
Indomethacin suppresses ESCC tumour growth in a PDX mouse model. (A) ESCC patient information. (B–D) PDX tumour size was plotted over 41 days (LEG74, *n* = 8/group), 26 days (LEG92, *n* = 12/group) or 13 days (LEG84, *n* = 10/group) to assess the effect of indomethacin. Vehicle or indomethacin (1 or 4 mg/kg) was administered intra‐gastrically. Tumour volumes were measured twice per week. (E) IHC staining of Ki67, ITGAV, ITGB3, p‐FAK, p‐PI3K, p‐AKT and p‐GSK3β in case LEG74. (F) The protein levels in case LEG74 tumours were determined by Western blotting. (G) The tumour inhibition rate was determined using the tumour weight. Each treatment group was compared with the control group. (H) ITGAV and ITGB3 protein levels in PDX tumours in cases LEG74, LEG92 and LEG84 were detected by Western blotting. The data show the tumour inhibition rate was significantly enhanced in groups with increased ITGAV expression than in those with reduced ITGAV expression. *: *p* < 0.05; **: *p* < 0.01; ***: *p* < 0.001. Data are presented as the mean value ± SD (error bar)

We next examined the effects of indomethacin in three different ESCC PDX models (Figure 6A). The results of the in vivo study showed that indomethacin treatment could significantly decrease tumour volume and weight in ESCC patient‐derived tumours (Figure 6B–D and Figure S6D–F). Indomethacin treatment did not reduce the mice body weight (Figure S6A–C). IHC staining results of tumours excised from the mice showed reduced Ki67, ITGAV, ITGB3, p‐FAK (Tyr925), p‐PI3K p85a (Tyr467), p‐AKT (Ser473), and p‐GSK3β (S9) protein levels in the indomethacin‐treated group compared to vehicle‐treated group (Figure 6E and Figure S6G). These results were confirmed by Western blotting (Figure 6F). Specifically, PDX tumours characterized by increased ITGAV protein levels (case LEG74) were more sensitive to indomethacin than those with low ITGAV protein levels (case LEG92 and LEG84) (Figure 6G and H). The enhanced inhibitory effect of indomethacin was not observed in tumours with increased COX protein levels (Figure S6H), suggesting that the inhibitory effect of indomethacin is dependent on ITGAV but not COX1/2 expression.

### 3.7 Indomethacin exerts stronger anti‐tumour activities than iRGD and cilengitide in vivo

**FIGURE 7:**
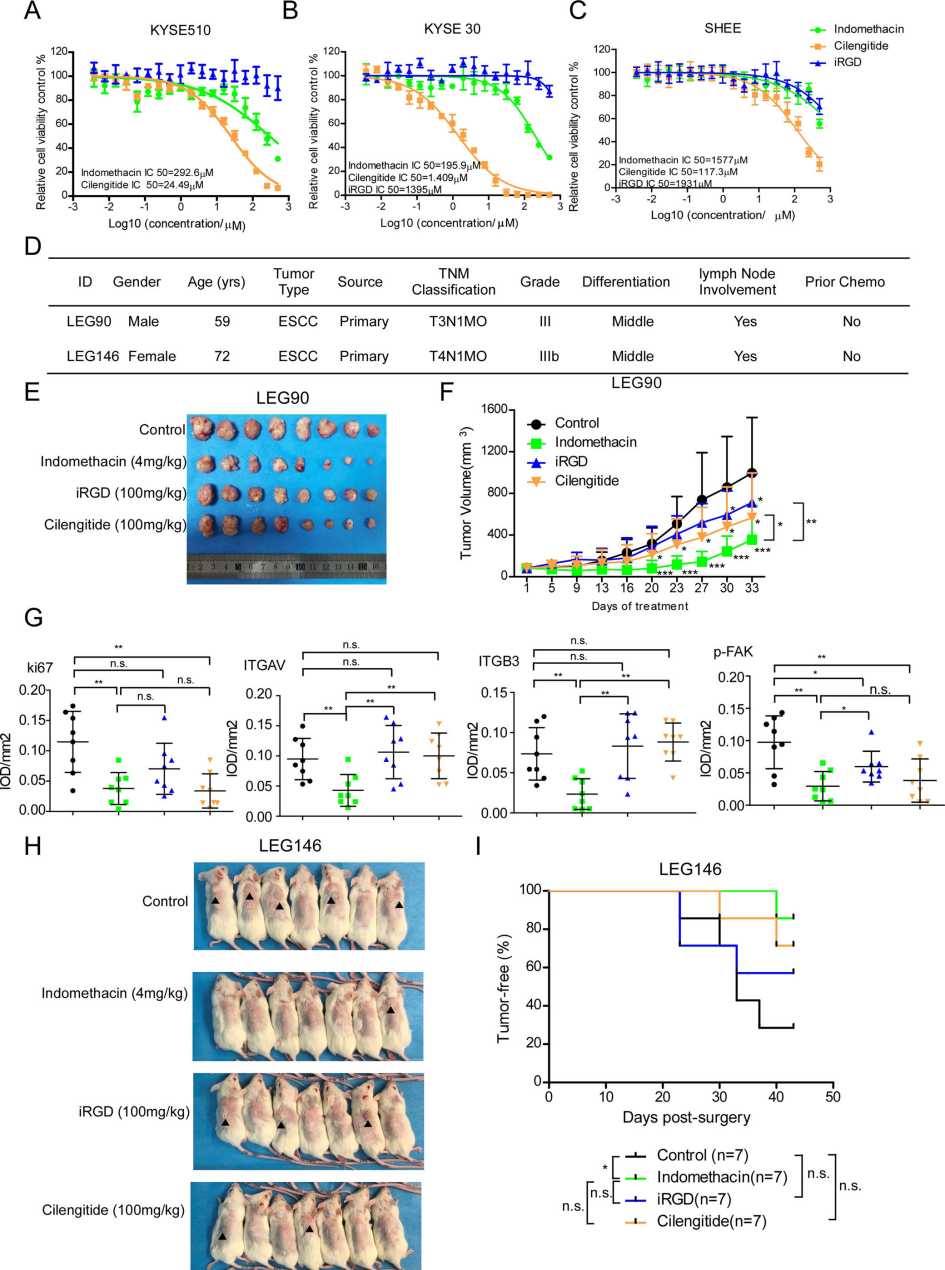
Indomethacin exerts stronger anti‐tumour activities than iRGD and cilengitide in vivo. (A–C) An MTT assay was performed 48 h after treating KYSE30, KYSE510 and SHEE cells with different concentrations of indomethacin, cilengitide and iRGD ranging between 0 and 500 µM for 48 h. (D) ESCC patient information. (E and F) PDX tumour size was plotted over 33 days (LEG90, *n* = 8/group) to assess the effect of indomethacin, iRGD and cilengitide. Vehicle or indomethacin (4 mg/kg) was administered by intra‐gastric administration. iRGD (100 mg/kg) and cilengitide (100 mg/kg) were administered by IP injection. Tumour volumes were measured twice per week. (G) IHC staining of Ki67, ITGAV, ITGB3, p‐FAK, p‐PI3K, p‐AKT and p‐GSK3β in case LEG90. (H). PDX tumours (case LEG 146) were inoculated into NOD‐SCID mice. After tumour volumes reached approximately 300 mm^3^, tumours were removed. Mice were randomized and then treated with indomethacin, iRGD or cilengitide for 43 days. Image shows a macroscopic view of mice harbouring recurrent tumours or tumour‐free animals at the end of the experiment. (I) Kaplan–Meier curve depicting recurrence‐free survival in mice treated with the indicated drugs or vehicle control. Recurrence was defined as the presence of a palpable tumour

We compared the inhibitory effect of indomethacin with known integrin inhibitors (iRGD and cilengitide) on KYSE510, KYSE30, and SHEE cell proliferation by MTT assay. Cells were treated with indomethacin, cilengitide, or iRGD at various concentrations from 0 to 500 µM for 48 h. The MTT results suggested that indomethacin‐treated KYSE510, KYSE30, and SHEE cells exhibited higher IC50 values compared to cells treated with cilengitide (Figure 7A and B). We did not observe a significant inhibitory effect in iRGD‐treated ESCC cell lines (Figure 7C). We next utilized a PDX model to investigate the potential anti‐tumour effects of indomethacin, iRGD, and cilengitide. The results indicated that each drug treatment group significantly inhibited ESCC tumour growth. Statistical analysis suggested that the inhibitory effect of indomethacin was more significant than that of iRGD and cilengitide (Figure 7E and F, Figure S7B). Treatment with indomethacin, iRGD, or cilengitide did not decrease the mice body weight (Figure S7A and C). We then performed IHC staining to detect ki67, ITGAV, ITGB3, and p‐FAK protein levels (Figure 7G). The results showed that indomethacin and cilengitide treatment decreased ki67 protein levels. Most interestingly, both ITGAV and ITGB3 were decreased in the indomethacin treatment group, but not in the iRGD and cilengitide treatment groups. However, treatment with indomethacin, iRGD, and cilengitide suppressed p‐FAK protein levels (Figure 7G and Figure S7D). These data indicated that treatment of ESCC tumours with iRGD or cilengitide inhibited integrin activity but did not contribute to integrin degradation. We then established an in vivo model to determine if indomethacin, iRGD, or cilengitide could affect ESCC recurrence. The results showed that indomethacin treatment prevented ESCC recurrence (Figure 7H and I).

### 3.8 Indomethacin suppresses TGFβ/SMAD2/3 signalling and enhances cancer immune responses

**FIGURE 8:**
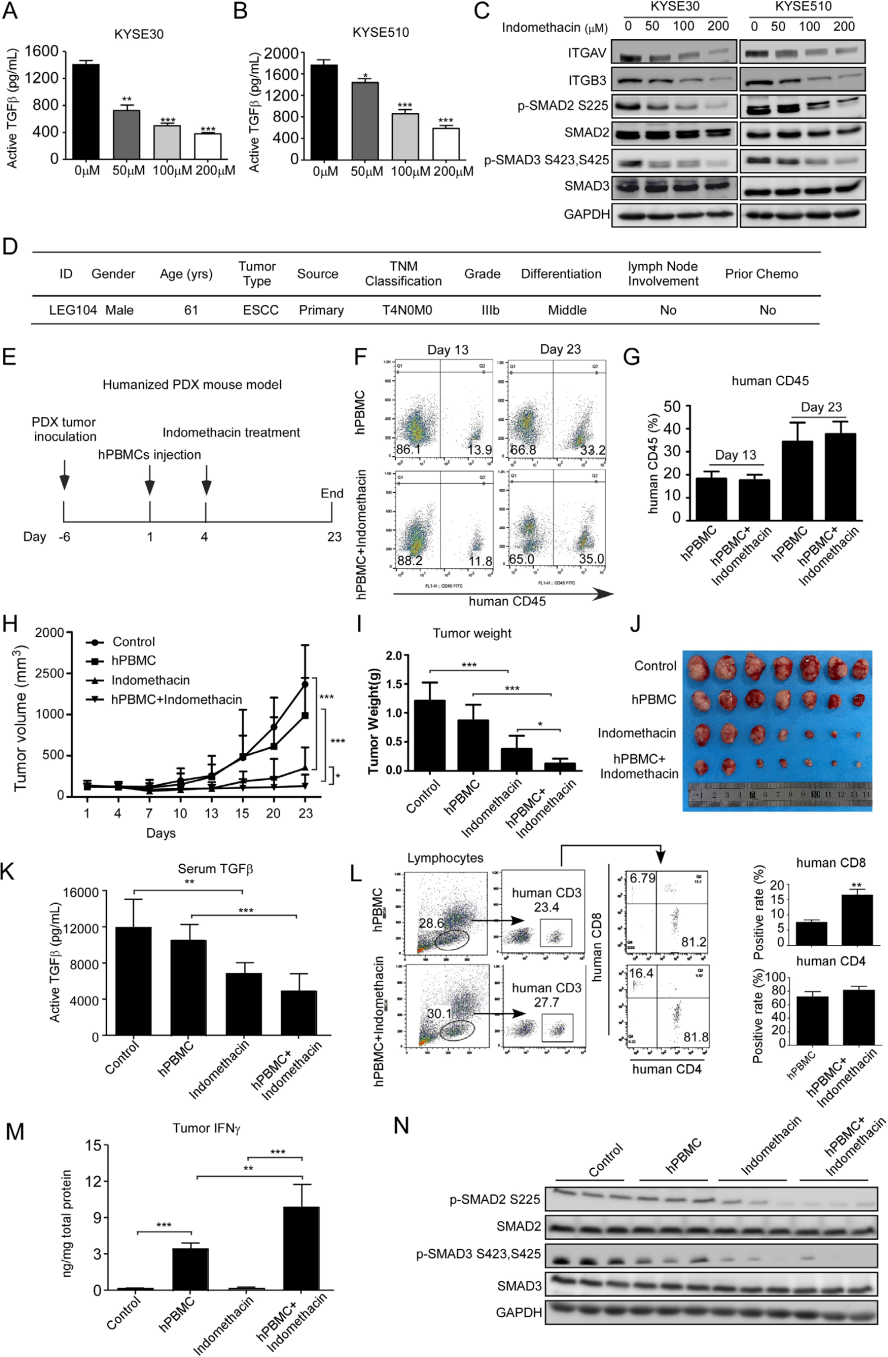
Indomethacin blocks TGFβ/SMAD2/3 pathway and enhances CD8^+^ T cell responses. (A and B) KYSE30 and KYSE510 cells were treated with indomethacin at the indicated concentrations for 48 h. The culture medium was collected for active TGFβ detection. (C) KYSE30 and KYSE510 cells were treated with various concentrations of indomethacin for 48 h. The indicated proteins were visualized by Western blotting. (D) ESCC patient information. (E) Time line for humanized PDX mouse model design. (F and G) Mice blood was harvested from the hPBMC injection groups at days 13 and 23 for human CD45 detection by flow cytometry. (H) PDX tumours (case LEG104) were inoculated into BRGSF mice and divided into four groups: (1) control, *n* = 7; (2) hPBMC, *n* = 7; (3) indomethacin, *n* = 7; and (4) hPBMC+indomethacin, *n* = 7. After 1 week, hPBMCs were injected into mice in groups 2 and 4. PDX tumours were measured and their size plotted over 23 days. Vehicle or indomethacin (4 mg/kg) was treated by intra‐gastric administration. (I and J) PDX tumours were excised and weighed at the end of the experiment. (K) Mice serum was harvested at the end of the experiment and human active TGFβ was determined by ELISA. (L) Mice blood was harvested and centrifuged. Cells were blocked and stained with CD3, CD4 and CD8 antibodies. Positive cell fractions were measured by flow cytometry. (M) Proteins were extracted from mice tumours and used for human IFNγ detection. (N) p‐SMAD2 and p‐SMAD3 protein levels in mice tumours were determined by Western blotting

Given that integrins play an important role in activating latent TGFβ complexes, we hypothesized that indomethacin may affect TGFβ activation. We measured the concentration of active TGFβ in the cell culturing medium after indomethacin treatment for 48 h. Results showed that the concentration of active TGFβ was decreased in the medium of the indomethacin treatment groups (Figure 8A and B). SMAD2/3 phosphorylation levels were also decreased upon indomethacin treatment (Figure 8C). These findings suggested that indomethacin may regulate immune cell response. Therefore, we detected the anti‐tumour activity of indomethacin in a humanized PDX mouse model (Figure 8E). Human CD45 levels in mice blood were determined 13 and 23 days after hPBMCs injection. We observed that human CD45 protein levels were increased more than 30% in the hPBMC injection group 23 days after hPBMCs injection (Figure 8F and G). Indomethacin treatment inhibited tumour growth in the non‐hPBMC injection group and the PBMC injection group. However, the tumour volumes in the hPBMC+indomethacin group were decreased compared to the indomethacin group (Figure 8H–J), suggesting that indomethacin treatment enhanced the immune response. Next, we determined the concentration of active TGFβ in the serum of mice from the control and treatment groups. The results suggested that indomethacin treatment prevented the activation of TGFβ (Figure 8K). Flow cytometry analysis demonstrated that indomethacin treatment increased the percentage of CD8^+^ T cells in mice blood, but there was no significant difference in human CD4 protein levels (Figure 8L). These findings are in agreement with previous studies that have suggested that the anti‐tumour effect exerted by TGFβ inhibitors is attributed to their effect on the CD8^+^ T cells populations.^40^, ^41^ It is well documented that IFNγ, which is secreted by immune cells, such as T cells, is critically important for the inhibition of cancer cell growth.^42^ Thus, we determined IFNγ protein levels in mice tumours. Importantly, we observed increased IFNγ protein levels in tumours after indomethacin treatment (Figure 8M). SMAD2/3 phosphorylation levels were also decreased upon indomethacin treatment (Figure 8N). These observations indicated that indomethacin inhibited the TGFβ/SMAD2/3 signalling axis and enhanced CD8^+^ T cell response in the tumour microenvironment.

**FIGURE 9:**
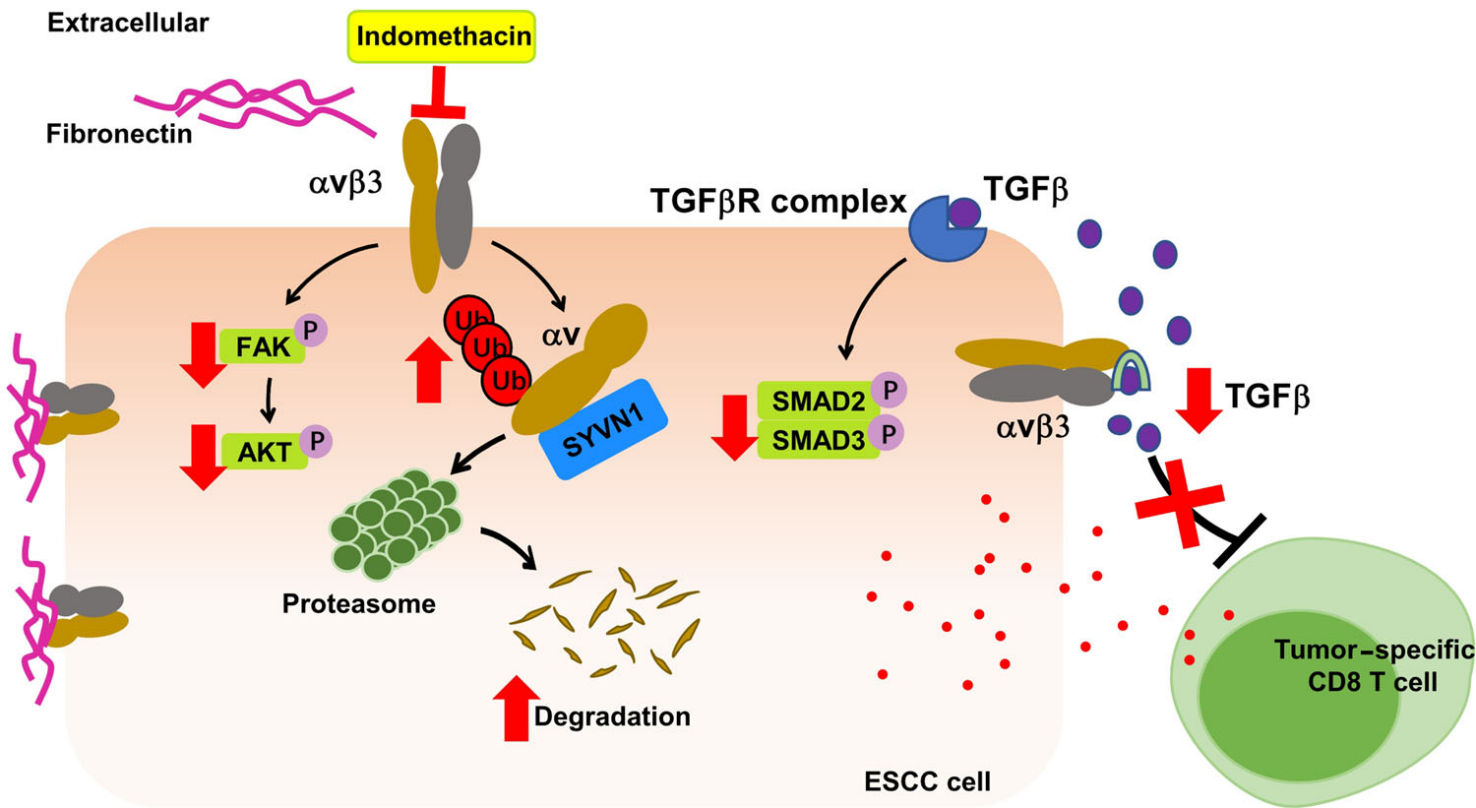
A schematic illustrating the suppression of cell proliferation and increased CD8^+^ T cell response upon treatment of ESCC tumours with indomethacin. Indomethacin directly binds to ITGAV, promotes the SYVN1‐mediated degradation of ITGAV and modulates the downstream FAK/PI3K/AKT and TGFβ/SMAD2/3 signalling pathways

## 4 DISCUSSION

ESCC is an aggressive cancer with a high mortality rate.^43^, ^44^ Despite recent advances in ESCC treatment modalities, the 5‐year overall survival rate is still relatively low due to the high risk of recurrence after primary treatment.^45^, ^46^ Thus, it is urgently necessary to find an effective strategy for preventing post‐operative ESCC recurrence. Currently, accumulating evidence have indicated that non‐steroidal anti‐inflammatory drugs, antimetabolism drugs, and natural compounds exert chemopreventive activities in multiple cancer types.^47^, ^48^, ^49^, ^50^ However, neither of these drugs or compounds have been approved for chemoprevention. In the present study, we identified that increased ITGAV expression was correlated with poor survival and was involved in ESCC cell proliferation. More importantly, we demonstrated that indomethacin suppressed ESCC growth and prevented recurrence through targeting integrin αvβ3.

Recent studies have shown that ITGAV is highly expressed in osteosarcoma, hepatocellular carcinoma, and oesophageal adenocarcinoma tissues compared to normal tissues.^51^, ^52^, ^53^ Additionally, inhibition of ITGAV has been shown to reduce tumour growth and metastasis in a variety of cancers.^54^, ^55^, ^56^ Herein, we observed that ITGAV was highly expressed in human ESCC tissues (Figure 1A–C) and that increased ITGAV levels were related with poor overall survival (Figure 1D). Knockdown of ITGAV effectively suppressed ESCC cell growth and invasion. In contrast, ESCC proliferation was increased after overexpression of ITGAV. Our results demonstrated that ITGAV plays an important role in ESCC proliferation.

Several current integrin inhibitors exhibit toxicity due to a combination of their on‐target and off‐target activities; these adverse effects may limit their clinical application in cancer therapy.^57^, ^58^ In order to identify affordable, low‐toxicity integrin inhibitors for use as potential chemoprevention agents, we performed a virtual drug screening and found that indomethacin may fit these criteria. Indomethacin was previously reported to reduce cancer cell growth by targeting COX.^30^, ^32^ In this study, we accrued evidence suggesting that indomethacin could bind to ITGAV through computational docking, SPR assay, and pulldown assay. Moreover, competition IP assay suggested that indomethacin can compete with ITGAV for its interaction with fibronectin. We also found that ITGAV protein levels were reduced after indomethacin treatment, and that MG132 prevented indomethacin‐induced ITGAV degradation (Figure 4C and D), suggesting that indomethacin‐ITGAV binding can promote ubiquitin‐dependent degradation of ITGAV. The ubiquitin‐proteasome system is important for regulating homeostasis between oncoproteins and tumour suppressors in cancer.^59^ E3 ubiquitin ligases play important roles in integrin ubiquitination.^60^ Blocking integrins with antibodies induces integrin degradation,^61^ suggesting that binding to integrins may promote the degradation of integrin receptors under specific conditions. Additionally, we identified that SYVN1 functions as an E3 ubiquitin ligase of ITGAV. Indomethacin‐ITGAV binding enhanced ITGAV ubiquitination and degradation, which is likely due to a conformational change within ITGAV that occurs upon indomethacin binding.^62^ It has been reported that adoption of an inactive conformation can enhance the ubiquitin‐mediated degradation of proteins.^63^ Integrin inactivators promote lysosomal degradation of integrins.^14^ Moreover, indomethacin treatment attenuated the integrin αvβ3 downstream FAK/PI3K/AKT/GSK3β signalling axis, which is strongly related with cell proliferation.^64^ Additionally, we found that indomethacin exerts anti‐tumour activities using the PDX mouse model, especially in tumours with increased ITGAV protein levels (Figure 6G and H), without causing significant body weight loss in mice. More importantly, when we compared the effects of indomethacin with known integrin inhibitors (iRGD and cilengitide) using the PDX mouse model, we found that indomethacin treatment showed a stronger anti‐tumour effect than treatment with either cilengitide or iRGD. Additionally, indomethacin treatment significantly prevented ESCC recurrence in the PDX mouse model.

Indomethacin is an anti‐inflammatory agent and could potentially affect immune cell function within the tumour microenvironment.^65^ Additionally, integrin αvβ3 promotes latent TGFβ activation,^18^ indicating that indomethacin may play an important role in TGFβ signalling. Indeed, indomethacin treatment suppressed the TGFβ/SMAD2/3 axis in vitro and in vivo. Studies have suggested that blockage of TGFβ signalling could enhance the proliferation of CD8^+^ T cells.^40^, ^66^ Herein, we found that indomethacin enhances anti‐tumour immune responses, including increasing CD8^+^ T cell population and IFNγ secretion in a humanized mouse model, implying indomethacin plays a vital role in the tumour microenvironment and may be a potential anti‐immunosuppressive strategy.

In this study, we administered 4 mg/kg/day of indomethacin for treatment to mice over the course of our in vivo experiments. This dose equates to 0.3 mg/kg/day in humans, meaning that the indomethacin level administered would correspond to a 21 mg/day for humans (weighing 70 kg). Notably, 50 mg indomethacin per day is typically used to treat pain,^67^ implying that low‐dose use of indomethacin may be an effective strategy for preventing ESCC recurrence. Clinical trials are recommended to verify the preventive effect of indomethacin on ESCC recurrence in humans.

In summary, our study establishes that ITGAV is highly expressed in ESCC and promotes tumour progression. Indomethacin binds to ITGAV, promotes ITGAV ubiquitination, and suppresses the integrin αvβ3/FAK/PI3K/AKT/GS3Kβ signalling axis. Moreover, indomethacin prevents the activation of TGFβ/SMAD2/3 signalling and enhances anti‐tumour immune responses (Figure 9). Importantly, indomethacin treatment suppresses ESCC tumour growth and recurrence, implying potential therapeutic application in the clinic.

## CONFLICT OF INTEREST

The authors declare that they have no conflict of interest.

## Supporting information

Supporting Information

Supporting Information

## Data Availability

The data that support the findings of this study are available from the corresponding author upon reasonable request.
