# Supplementary material for: Targeting integrin αvβ3 with indomethacin inhibits patient‐derived xenograft tumour growth and recurrence in oesophageal squamous cell carcinoma
Source: Clin Transl Med. 2021 Oct 28;11(10):e548. doi: 10.1002/ctm2.548 (PMC8552524; doi:10.1002/ctm2.548)

**Supplementary Materials: Targeting integrin αvβ3 with indomethacin inhibits patient-derived xenograft tumor growth and recurrence in esophageal squamous cell carcinoma**

**Supplementary Figure Legends**

Figure S1. Integrin family member mRNA and protein levels in ESCC and normal tissues.

(A) mRNA expression levels of ITGA1-11, ITGAV, and ITGB1-8 in ESCC and normal tissues are obtained from the TCGA database. **: *p* < 0.01; ***: *p* < 0.001. Data are presented as the mean value ± SD (error bar). (B) The protein levels of ITGAV, ITGA2, ITGA3, ITGA4, ITGA6, ITGA11, ITGB1, ITGB3, ITGB5, ITGB6, and ITGB8 in ESCC tumors and adjacent tissues were assessed by Western blotting. Table: number of patients with high levels of the indicated genes/number of total detectable patients.

Figure S2. Downregulation of integrin αvβ3 blocks PI3K/AKT signaling and suppresses ESCC tumor growth.

(A) The protein levels of p-FAK, FAK, p-PI3K, PI3K, p-AKT, and AKT in shMock and shITGAV cells were determined by Western blotting. (B) The protein levels of p-FAK, FAK, p-PI3K, PI3K, p-AKT, and AKT in vector- and ITGAV-overexpressing cells were determined by Western blotting. (C, D) The protein levels of integrin β subunits were assessed by Western blotting after ITGAV knockdown or overexpression. (E-F). *Nu/nu* mice were subcutaneously inoculated with 1×10^7^ KYSE30 or KYSE510 cells into the right flank. The mice were sacrificed when the tumor volumes reached approximately 1 cm^3^. After KYSE30 or KYSE510 CDX mice were euthanized, the tumors were excised and weighed. (G) Tumor tissues were subcutaneously inoculated into the right flank of NOD/SCID mice. After allowing the tumor to grow (~200 mm^3^), lentivirus was injected at multiple tumor sites. The tumors were excised and weighed when their volume reached approximately 1 cm^3^. *: *p* < 0.05; **: *p* < 0.01; ***: *p* < 0.001. Data are presented as the mean value ± SD (error bar).

Figure S3. Indomethacin does not directly bind to ITGB1-8.

KYSE30 cell lysates were mixed with 50 μL of disulfiram (A) or indomethacin (B)-conjugated Sepharose 4B beads or DMSO-conjugated Sepharose 4B beads in reaction buffer and the indicated pulled-down proteins were detected by Western blotting. (C) KYSE30 cells were treated with 200 μM indomethacin for the indicated times. The protein levels of ITGB1,5,6,8 subunits were assessed by Western blotting.

Figure S4. SYVN1 was identified as an E3 ubiquitin ligase of ITGAV.

(A) UbiBrowser predicted the potential E3 ubiquitin ligases of ITGAV. The top 12 predicated E3 ubiquitin ligases of ITGAV are shown. (B) KYSE 30 cells processed for an IP assay with IgG or anti-ITGAV antibodies. Interactions between ITGAV and the indicated proteins were detected by Western blotting.

Figure S5. Indomethacin induces G1-phase arrest and apoptosis in KYSE 30 and KYSE 510 cells.

(A) An MTT assay was performed after treating SHEE cells with various concentrations of indomethacin for 24, 48, 72, or 96 h. Three independent experiments were performed. Data are presented as the mean value ± SD (error bar). (B-C) Cells were seeded in 6-cm dishes and incubated for 12 h, followed by treatment with the indicated concentrations of indomethacin for 48 h. The cells were then harvested for cell cycle analysis. (D-E) Cells were treated with various concentrations of indomethacin for 72 h and the distribution of apoptotic cells was detected by flow cytometry. (F) COX1/2 protein levels in KYSE30, KYSE70, KYSE140, KYSE150, KYSE410, KYSE450, and KYSE510 ESCC cell lines was examined by Western blotting. (G-H) The correlation between the protein levels of COX1 (*left panel*)/2 (*right panel*) and the cell growth inhibition rate in various ESCC cell lines. n.s., no significant difference.

Figure S6. Indomethacin suppresses ESCC tumor growth in a PDX mouse model. (A-C) Mice were orally administered vehicle or indomethacin at 1 or 4 mg/kg every day for 41 days in case LEG74, every 26 days in case LEG92, and every 13 days in case LEG84. The body weight of mice in both treatment groups was measured twice a week. (D-F) PDX tumors were excised from the vehicle and indomethacin treatment groups and weighed at the end of the experiment. Tumor weight was significantly reduced in the indomethacin treatment group compared to the vehicle treatment group. *: *p* < 0.05; **: *p* < 0.01; ***: *p* < 0.001. Data are presented as the mean value ± SD (error bar). (G) Indomethacin treatment decreased the protein levels of Ki67, ITGAV, ITGB3, p-FAK, p-PI3K, p-AKT, and p-GSK3β in PDX tumors. IHC and H&E staining were performed in tumors from case LEG74. Representative images are shown (100× magnification). Scale bar: 50 μm. (B) COX1 and COX2 protein levels in ESCC PDX tumors were detected by Western blotting.

Figure S7. Indomethacin exerts stronger anti-cancer properties than iRGD and cilengitide *in vivo*.

(A) Body weight in mice from the vehicle and indomethacin treatment groups was measured twice a week (case LEG90). Vehicle or indomethacin (4 mg/kg) was administered intragastrically. iRGD (100 mg/kg) and cilengitide (100 mg/kg) were administered by IP injection (case LEG90). (B) PDX tumors were excised from each group and weighed at the end of the experiment. (C) Body weight changes within the ESCC recurrence model (case LEG146). Body weight in mice from the vehicle and indomethacin treatment groups was measured twice a week. (D) IHC and H&E staining was performed in tumors from case LEG146. The protein levels of Ki67, ITGAV, ITGB3, and p-FAK in each group were assessed by IHC staining. Representative images are shown (100× magnification). Scale bar: 50 μm.

Figure S8. Indomethacin treatment does not affect the body weight in mice.

Body weight in mice from the vehicle and indomethacin treatment groups was measured twice a week (case LEG269).

Supplementary Table S1

Showing the docking score of top 20 FDA-approved drugs to the ITGAV

| **Drug Name** | **CAS** | **Docking score** | **Diease** |
| --- | --- | --- | --- |
| Pemetrexed acid | 137281-23-3 | -7.241 | Malignant pleural mesothelioma |
| Oxaliplatin | 61825-94-3 | -6.33 | Colorectal cancer |
| Gemcitabine | 95058-81-4 | -6.298 | Advanced Pancreatic cancer |
| Lapatinib ditosylate monohydrate | 388082-78-8 | -6.06 | Breast Cance |
| Sorafenib tosylate | 475207-59-1 | -5.718 | Advanced renal cell carcinoma |
| Bendamustine hydrochloride | 3543-75-7 | -5.361 | Chronic Lymphocytic Leukemia |
| **Indomethacin** | **53-86-1** | **-5.34** | **Pain related to inflammation** |
| Sorafenib tosylate | 475207-59-1 | -5.326 | Advanced renal cell carcinoma |
| Sorafenib | 284461-73-0 | -5.326 | Hepatocellular carcinoma |
| Enzastaurin | 170364-57-5 | -5.318 | Glioblastoma multiform |
| Quinestrol | 152-43-2 | -5.301 | Contraceptive drug |
| Hydroxy Camptothecine | 64439-81-2 | -5.301 | Multiple cancer |
| 10-Hydroxycamptothecin | 19685-09-7 | -5.301 | Multiple cancer |
| Imiquimod | 99011-02-6 | -5.301 | Superficial basal cell carcinoma |
| EPZ6438 | 1403254-99-8 | -5.193 | Lymphoma |
| Bosutinib | 380843-75-4 | -5.051 | Leukemia |
| Dinaciclib | 779353-01-4 | -4.965 | CDK inhibitor |
| **Disulfiram** | **97-77-8** | **-4.93** | **Anti-alcoholic drug** |
| Pacritinib | 937272-79-2 | -4.915 | Myelofibrosis |
| Fosbretabulin Disodium | 168555-66-6 | -4.744 | Vascular-disrupting agent |


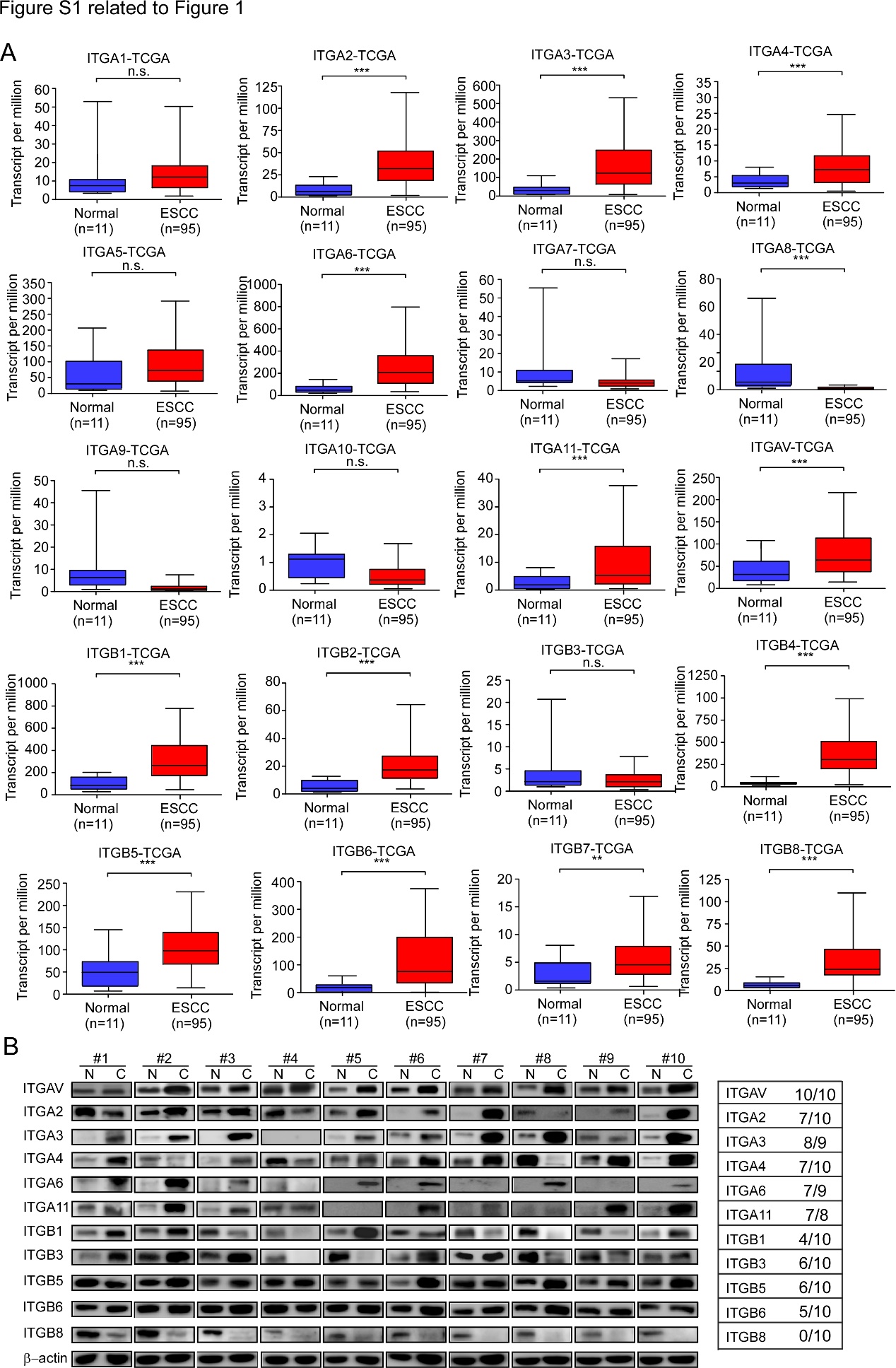


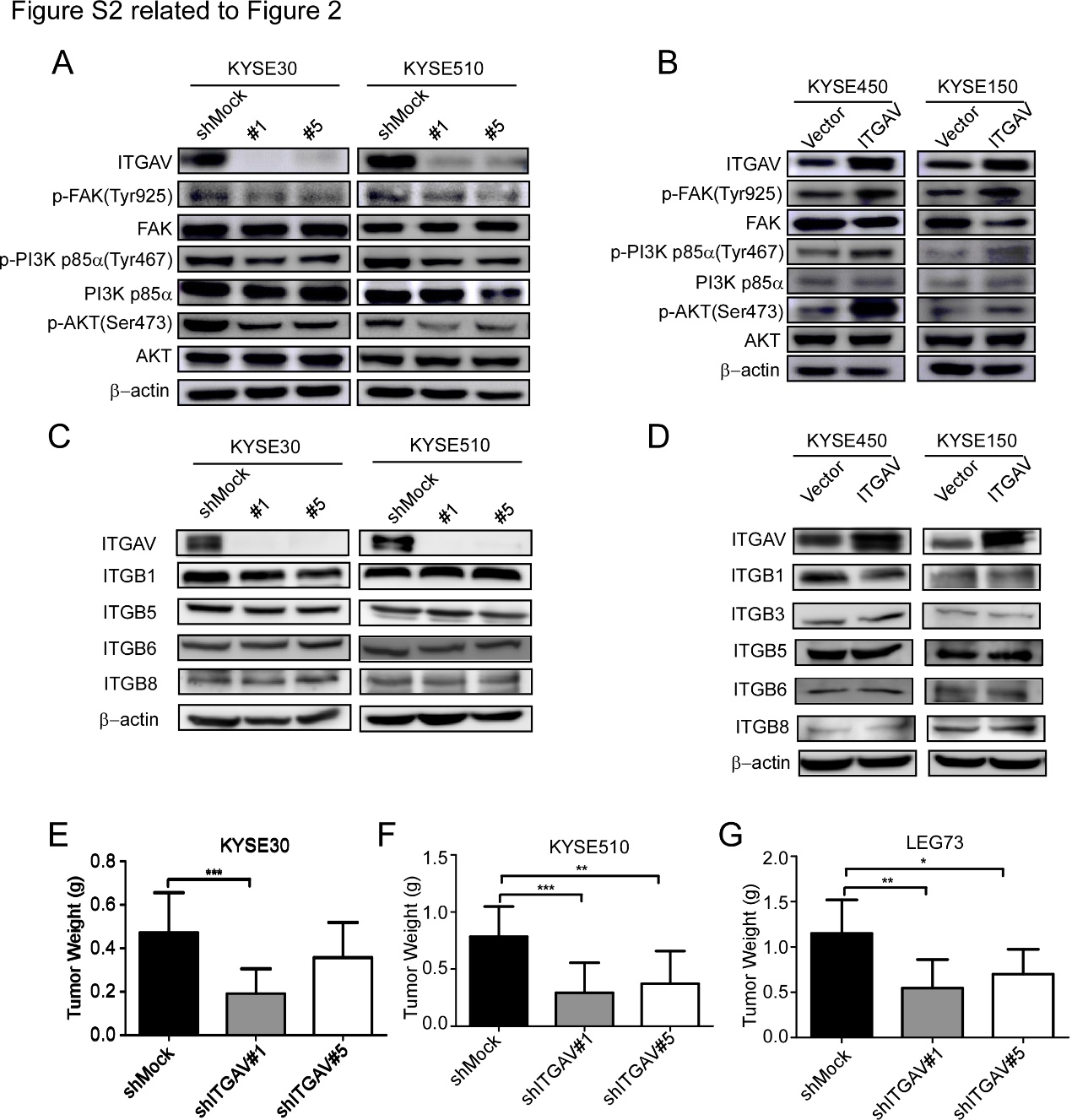


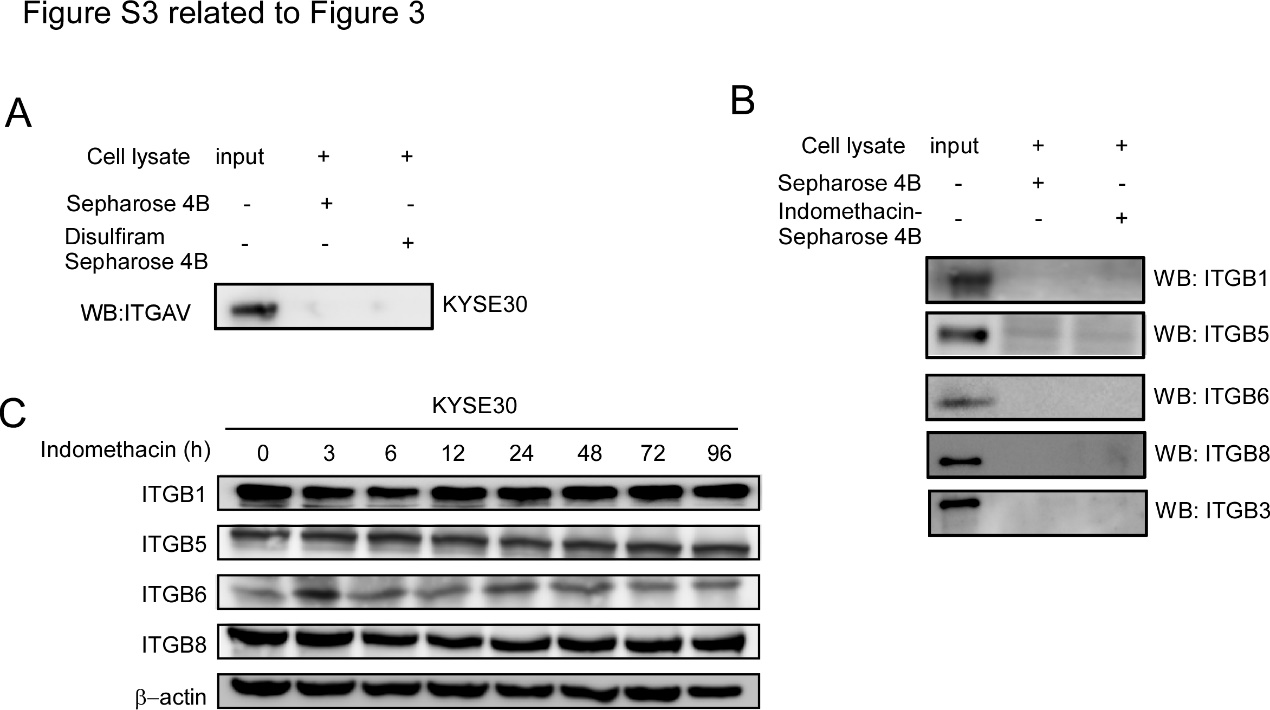


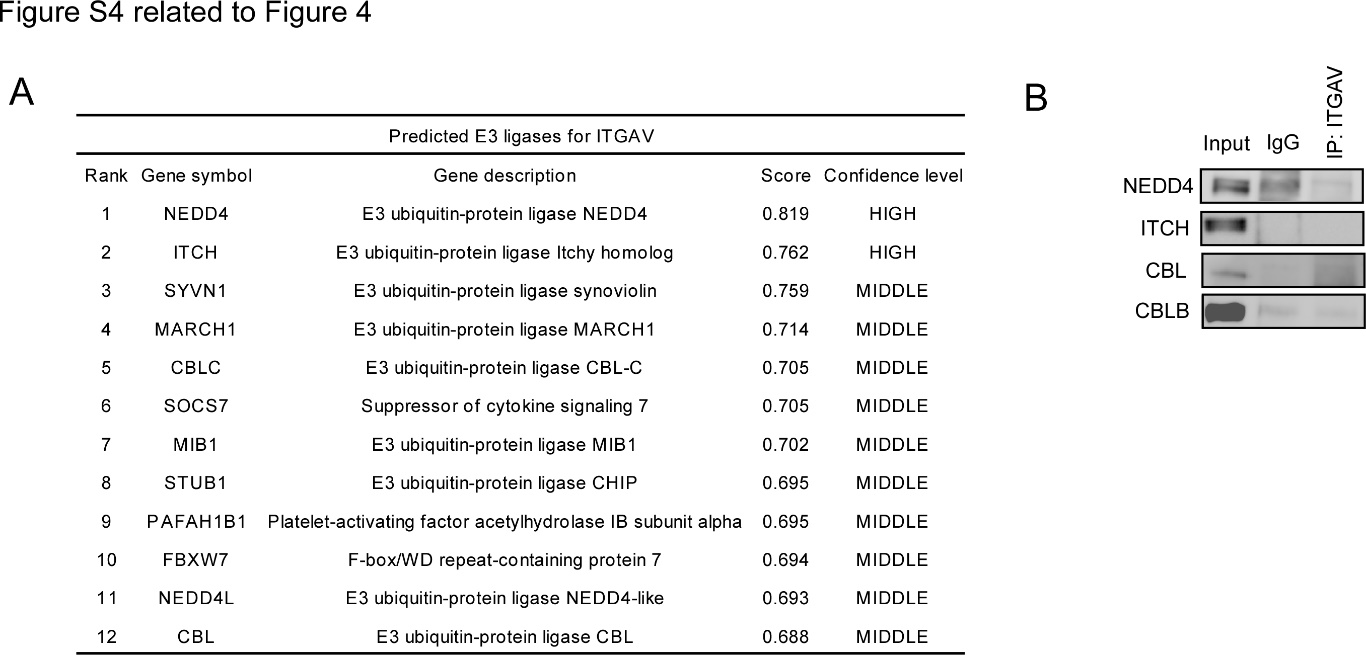

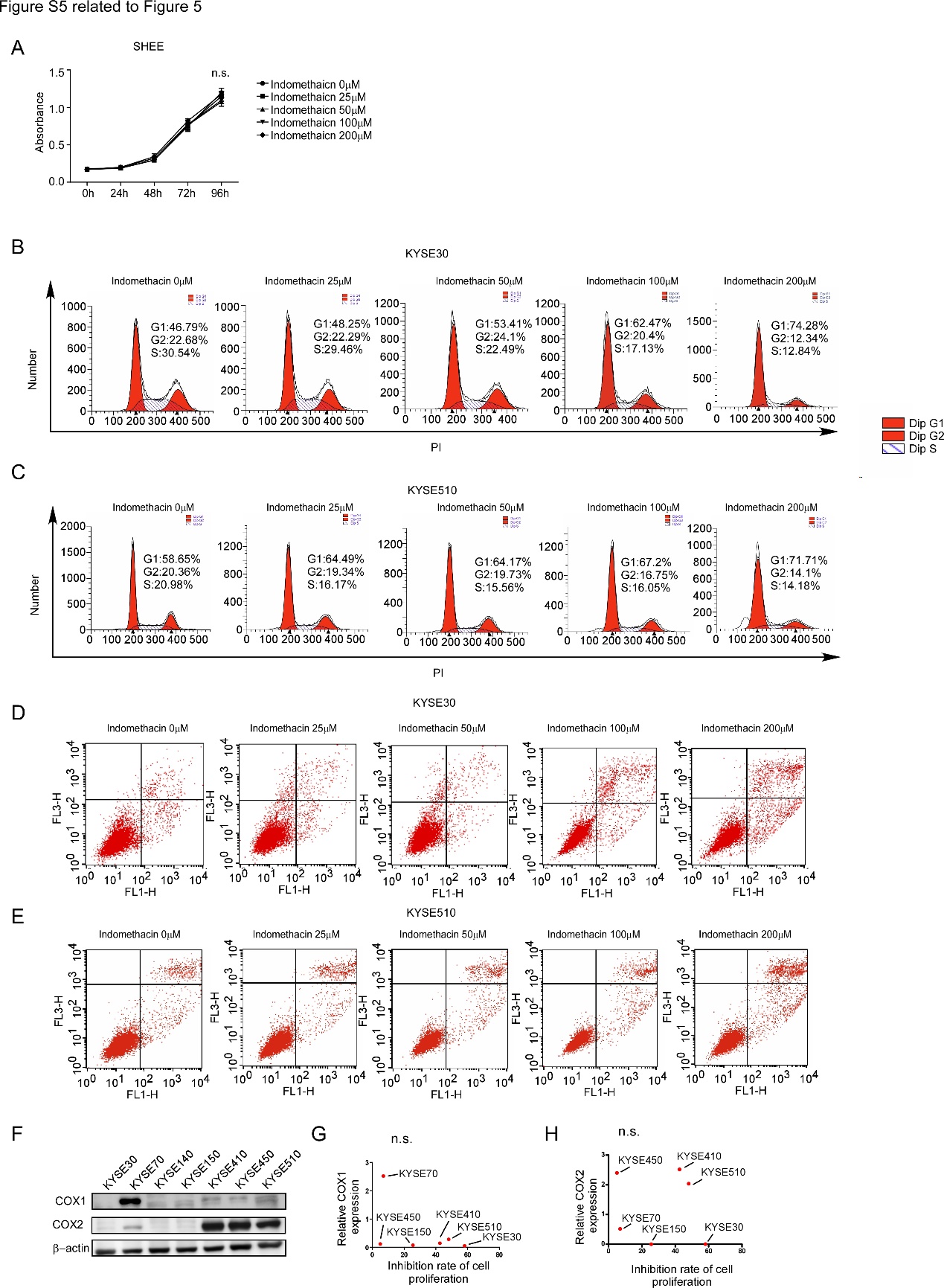

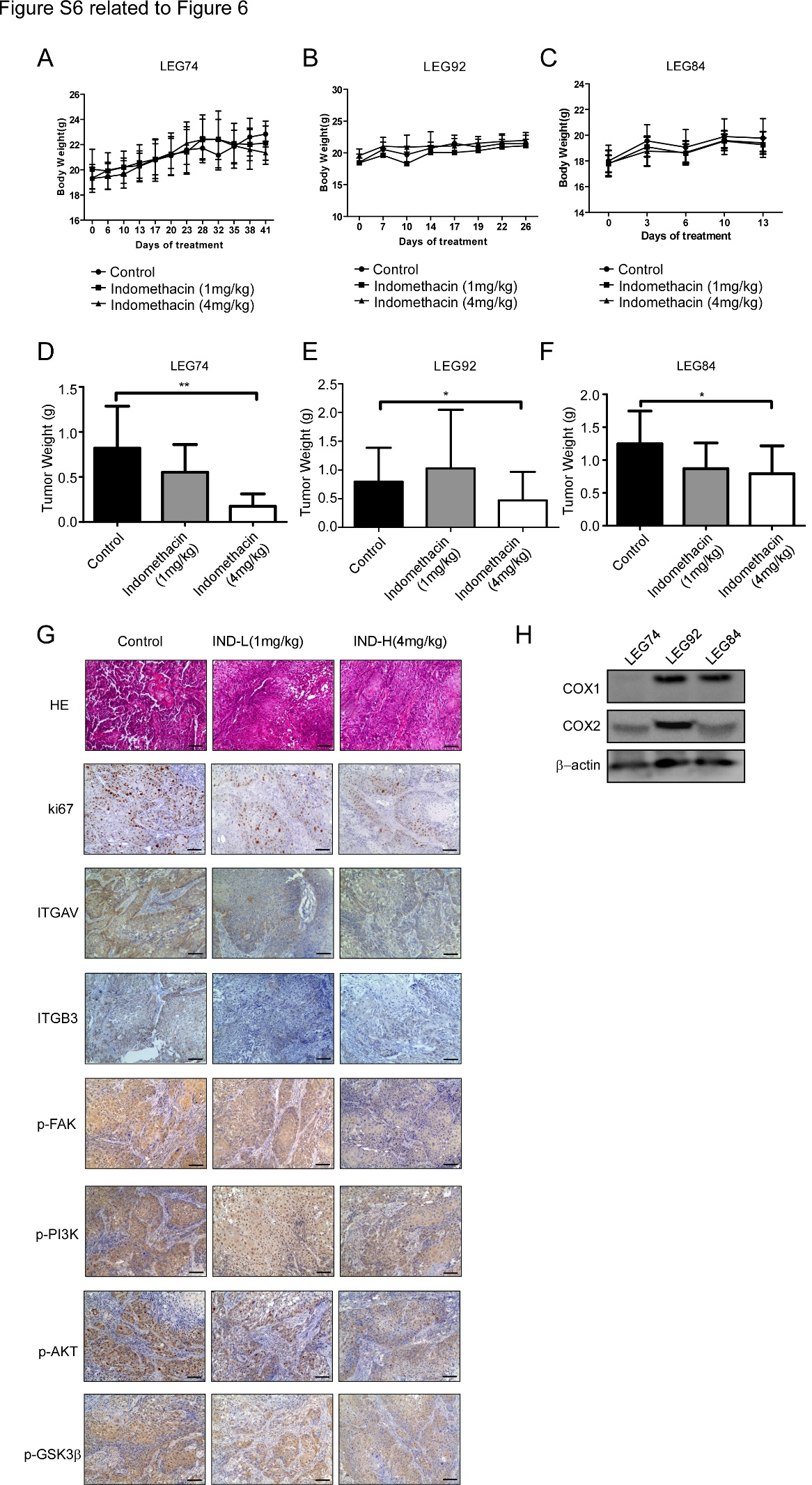

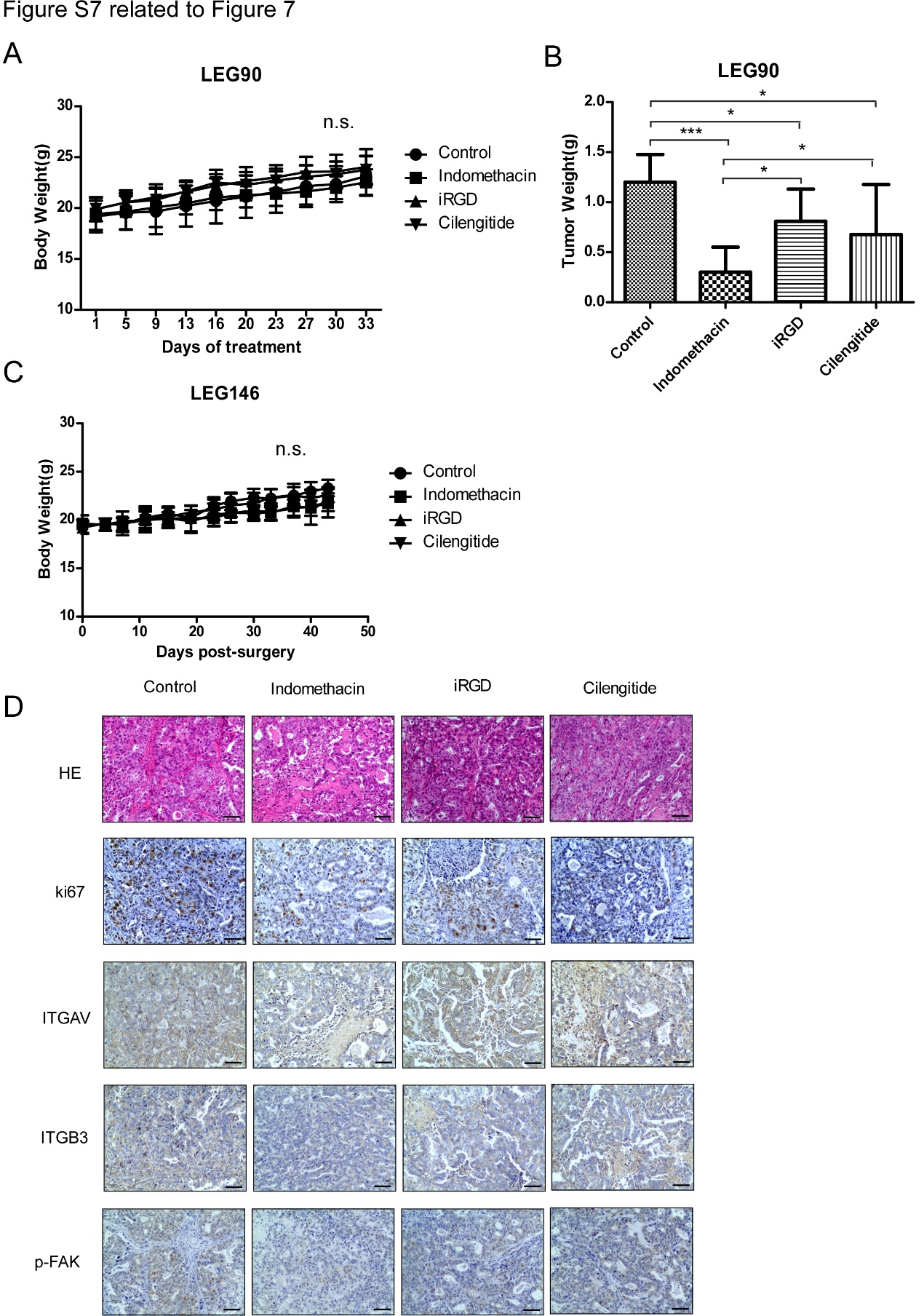

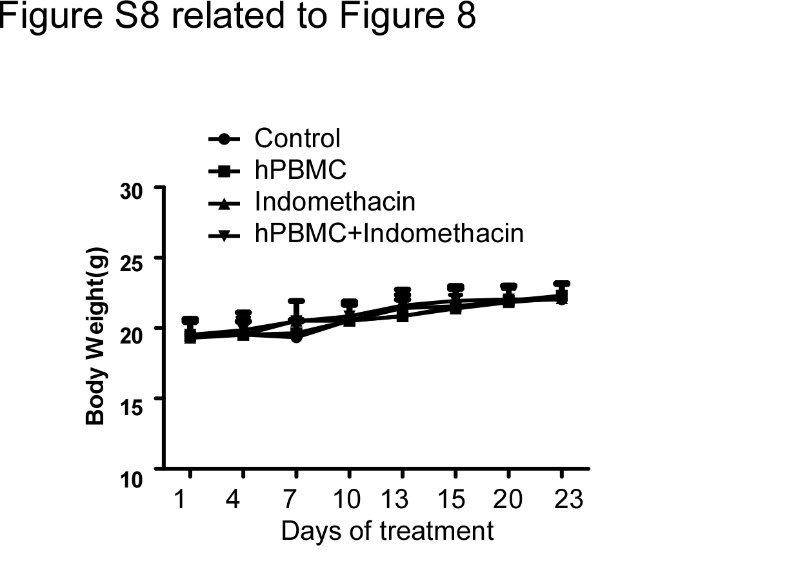

Supplement: Supplementary file 1 — Supporting Information [file CTM2-11-e548-s002.docx]
